# Handling the Hurdles on the Way to Anti-tuberculosis Drug Development

**DOI:** 10.3389/fchem.2020.586294

**Published:** 2020-11-19

**Authors:** Pedro F. Dalberto, Eduardo V. de Souza, Bruno L. Abbadi, Christiano E. Neves, Raoní S. Rambo, Alessandro S. Ramos, Fernanda S. Macchi, Pablo Machado, Cristiano V. Bizarro, Luiz A. Basso

**Affiliations:** Centro de Pesquisas em Biologia Molecular e Funcional (CPBMF) and Instituto Nacional de Ciência e Tecnologia em Tuberculose (INCT-TB), Pontifícia Universidade Católica do Rio Grande do Sul (PUCRS), Porto Alegre, Brazil

**Keywords:** tuberculosis, antimycobacterial, medicinal chemistry, drug screening, drug development, target, therapy, gene validation

## Abstract

The global epidemic of tuberculosis (TB) imposes a sustained epidemiologic vigilance and investments in research by governments. *Mycobacterium tuberculosis*, the main causative agent of TB in human beings, is a very successful pathogen, being the main cause of death in the population among infectious agents. In 2018, ~10 million individuals were contaminated with this bacillus and became ill with TB, and about 1.2 million succumbed to the disease. Most of the success of the *M. tuberculosis* to linger in the population comes from its ability to persist in an asymptomatic latent state into the host and, in fact, the majority of the individuals are unaware of being contaminated. Even though TB is a treatable disease and is curable in most cases, the treatment is lengthy and laborious. In addition, the rise of resistance to first-line anti-TB drugs elicits a response from TB research groups to discover new chemical entities, preferably with novel mechanisms of action. The pathway to find a new TB drug, however, is arduous and has many barriers that are difficult to overcome. Fortunately, several approaches are available today to be pursued by scientists interested in anti-TB drug development, which goes from massively testing chemical compounds against mycobacteria, to discovering new molecular targets by genetic manipulation. This review presents some difficulties found along the TB drug development process and illustrates different approaches that might be used to try to identify new molecules or targets that are able to impair *M. tuberculosis* survival.

## Introduction

Human tuberculosis (TB), mainly caused by the bacterial pathogen *Mycobacterium tuberculosis*, is a chronic, infectious-contagious, and deadly disease that has reached pandemic proportions. Typically, the active form of the disease affects the lungs (pulmonary TB) but in ~25% of patients (immunocompromised patients or children) mycobacteria may enter the blood stream and infect other parts of the body such as the pleura, the meninges, the lymphatic system, the genitourinary system, bones, and joints (Smith, 2003). The World Health Organization (WHO) estimated that, globally, 10 million people felt ill with TB in 2018 (World Health Organization, 2019). Approximately 1.2 million TB deaths occurred in the same year in HIV-negative and 251,000 in HIV-positive patients (World Health Organization, 2019). The highest burden of TB infection is in men (aged ≥15 years that accounts for 57% of all cases), followed by women (32%) and children (aged <15 years that accounts for 11%) (World Health Organization, 2019). All Member States (194) reported cases of TB to WHO in 2018, and the Southeast Asia (44%), Africa (24%), Western Pacific (18%), and Eastern Mediterranean (8%) regions accounting for most of them (World Health Organization, 2019).

Effective drug treatments were first developed in the 1940s. The currently recommended treatment for cases of drug-susceptible TB disease is a 6-month regimen of four first-line drugs (isoniazid, rifampicin, ethambutol. and pyrazinamide) (Furin et al., 2019; World Health Organization, 2019). The Global TB Drug Facility supplies a complete 6-month course for about US$ 40 per person. Success rates of at least 85% for cases of drug-susceptible TB are regularly reported to WHO for treatments with first-line drugs. In 2018, there were ~500,000 new cases of rifampicin-resistant TB (RR-TB), 78% of which were infected with multi-drug resistant (MDR-TB) strains (resistant to rifampicin and isoniazid). Globally the treatment success rate for patients infected with MDR/RR-TB is 56%. It is estimated that in 2018, an average of 6.2% of MDR-TB patients were infected with extensively drug-resistant strains of *M. tuberculosis* (XDR-TB) (World Health Organization, 2019), which is defined as MDR-TB plus resistance to at least one of the fluoroquinolones (such as ofloxacin, levofloxacin or moxifloxacin) and one of the injectable second-line agents used in MDR-TB treatment regimens (amikacin, capreomycin, or kanamycin). In 2009, Valayati and colleagues identified TB strains in Iran that were resistant to all first and second line drugs (which includes isoniazid, rifampicin, ethambutol, pyrazinamide, ethionamide, para-aminosalicylic acid, cycloserine, ofloxacin, amikacin, ciprofloxacin, capreomycin, kanamycin), and termed these strains as totally drug-resistant TB (TDR-TB) or extremely drug resistant TB (XXDR-TB) (Velayati et al., 2009). These strains had previously been isolated from Italian patients (Migliori et al., 2007). However, these terms are yet to be recognized by the WHO, and further studies to characterize these highly resistant strains and to propose novel effective therapies are still needed. It is estimated that ~10% of all MDR-TB strains are TDR-TB, but many laboratories that are in countries with high TB burden lack the capacity of detecting and isolating these strains properly. Therefore, it is believed that the number of TDR-TB cases are underestimated and might account for 10% of all MDR-TB strains (Velayati et al., 2018). The readers interested in a thorough description of the chemical structure, mode of action, and mechanism of resistance to anti-TB agents currently recommended by the WHO to be used for treatment of susceptible and drug-resistant strains are referred to the excellent review published by Mabhula and Singh (2019).

Treatments for people infected with RR-TB and MDR-TB are longer, require the use of more expensive drugs (≥US$ 1,000 per person), and are considerably more toxic. The side effects caused by treatments for resistant cases can vary from mild effects, such as complaints of gastrointestinal disturbance (nausea, anorexia, diarrhea, vomiting, and abdominal pain), headaches, and insomnia, to severe effects, such as psychosis, seizures, heart rhythm disorder (prolongation of the QT interval), hypothyroidism, arthralgia, nephrotoxicity, hepatotoxicity, ototoxicity, and dermatologic adverse effects (Ramachandran and Swaminathan, 2015). The effectiveness of therapies with first- and second-line drugs depends on the commitment of the patient to completing the treatment, if the infection is in the active form, and the sensitivity of the carried strain to the administered drugs. However, many infected individuals do not adhere to treatment due to the severe side effects, treatment complexity with large number of different drugs and doses, and especially the long duration (six to 12 months, up to 2 years, depending on the strain). The main consequence of poor adherence by patients and prescription of erroneous treatment regimen is the maintenance of the disease in the infected individual, which results in continuous disease transmission and spreading of resistant strains (Furin et al., 2019). Although the immune system of some individuals can promptly eradicate the infection, most individuals contain the bacteria in a latent state. It is estimated that ~1.7 billion people holds latent TB infection (LTBI), which is characterized by patients that are infected with *M. tuberculosis* but have no symptoms and do not transmit the infection to others. Individuals having latent TB represent a considerable reservoir of the bacilli for future outbreaks of this disease, and efforts should be pursued to diagnose and treat these patients (Ehrt et al., 2018; World Health Organization, 2019). Four options for treatment of LTBI are available: a weekly dose of rifapentine and isoniazid for 3 months; a daily dose of rifampicin plus isoniazid for 3 months; a daily dose of rifampicin for 3–4 months; and a daily dose of isoniazid for at least 6 months (Furin et al., 2019). Therefore, new chemical agents should be developed to allow affordable, simpler and faster TB treatment of pan-sensitive strains to increase patient adherence, be effective against drug-resistant strains, cut the risk of progression to active TB in latently infected individuals, and, ideally, result in sterilization of bacilli from infected patients (defined as clearance of the entire microbial population from all tissues of the host).

The two main interventions employed to prevent new infections of *M. tuberculosis* and their progression to active TB are treatment of LTBI and vaccination of children with the bacille Calmette-Guérin (BCG) vaccine, which was developed by serial *in vitro* passages of *Mycobacterium bovis* to achieve non-pathogenicity. TB endemic countries use the vaccine to ensure protection of children against severe forms of the disease, such as meningitis TB and miliary TB (Pang et al., 2016). However, its efficacy against pediatric pulmonary TB ranges from no protection to very high protection (0–80%) (Pang et al., 2016). Moreover, vaccination against sensitive and drug-resistant strains of *M. tuberculosis* is not effective in preventing pulmonary TB in adults, either before or after exposure to TB infection. The discrepancies in protection afforded by BCG may be attributed to the following: (i) over-attenuation of the BCG strain, resulting in an inefficient immune response; (ii) different types of BCG strains; (iii) genetic differences between human populations, which may result in elimination of BCG in some individuals before the development of a protective immune response; (iv) inefficient cold chain maintenance of BCG; and (v) the exposure of some children to environmental non-tuberculous mycobacteria, leading to tolerance (Pang et al., 2016). Even after almost 100 years of use, the BCG vaccine has been the focus of recent studies to improve its protection. Current strategies range from alternative routes of BCG administration (Darrah et al., 2020) to genetic manipulation (Festjens et al., 2019). Intravenous immunization with BCG in macaques was shown to induce more T cells antigen-responses when compared to the common intradermal administration route, protecting these animals against *M. tuberculosis* challenge after 6 months of vaccination (Darrah et al., 2020). A live attenuated *sapM* mutant of BCG Pasteur strain that lacks protein expression of secreted acid phosphatase (SapM) showed a more effective innate control when compared with wild type BCG (Festjens et al., 2019).

New strategies are thus urgently needed to prevent and treat TB pandemic worldwide, with special attention to drug-resistant strains. Several peculiarities of the bacilli biology and the TB pathology, together with old challenges of the pharmacology field, are recognized to complicate the development of new effective therapies, and they should all be taken into consideration during the long journey of the TB drug development process.

## Heterogeneous Milieu of *Mycobacterium Tuberculosis* Represents a Challenge for the Development of New Antimycobacterial Agents

### Diverse Niches of TB Pathology

*M. tuberculosis* infection initiates with inhalation of air-born droplets containing viable bacilli exhaled by a patient with active TB disease. Bacilli are taken up by phagocytic cells and transported across the alveolar epithelium into the lung (Dartois, 2014). A hallmark of *M. tuberculosis* infection is the establishment of chronic disease, in which bacilli in alveolar macrophages recruit additional macrophages and other immune cells and induce formation of granulomas, which are organized immune complexes of differentiated macrophages, T lymphocytes, some B lymphocytes, dendritic cells, neutrophils, fibroblasts, and extracellular matrix components (Cosma et al., 2003). A single patient may present heterogeneous structures of granuloma. Granulomas evolve morphologically during the course of TB infection, with the formation of areas of necrosis called caseum (regions of acellular debris) and the deposition of fibrin and calcium (related to healing or healed lesions as it only occasionally contains live bacteria) (Cosma et al., 2003). Human granulomas can caseate, and rupture of liquefied caseum containing bacteria (dead and viable) allows for person-to-person transmission of viable bacilli, and ensuing pulmonary tuberculosis (Cosma et al., 2003). On one hand, RNA-RNA *in situ* hybridization for mycobacterial targets (an indicator of live bacteria) in granulomas from resected lung of humans with caseus tuberculosis suggested that RNA expression was only found associated with macrophages and giant cells in non-necrotic zones (Fenhalls et al., 2002). On the other hand, an *ex vivo* assay has been developed to measure the cidal activity of anti-TB drugs against bacilli (non-replicating, viable, and first- and second-line drug resistant) present in cavity caseum obtained from rabbits with active disease (Sarathy et al., 2018). The latter suggests that viable bacilli are present in the necrotic zone of granulomas and are largely non-replicating and exhibit extreme tolerance to many first- and second-line TB drugs. In the various niches in which *M. tuberculosis* resides in a granuloma, differing environmental characteristics may be encountered by the bacilli such as hypoxia (Via et al., 2008), nutrient starvation (Nyka, 1974), low pH (Cosma et al., 2003), and reactive oxygen and nitrogen species (Voskuil et al., 2011). These environmental stresses have been shown to induce a non-replicating, phenotypically drug-resistant state *in vitro* (Wayne and Hayes, 1996; Gold et al., 2015).

### Examples of *in vitro* Models for Non-replicating and Replicating Bacilli

Subpopulations of non-replicating bacilli have been proposed to contribute to the lengthy time course of TB treatment and to represent a reservoir from which drug-resistant bacteria emerge (Katsuno et al., 2015). Although non-replicating bacilli has been invoked to underlie latent infections, it has been pointed out that this proposal should be viewed with caution (Cosma et al., 2003). Two (non-mutually exclusive) proposals have been put forward to explain the need for the long and intensive TB therapy: (1) bacterial populations sequestered in remote lesion compartments which antibiotics fail to reach therapeutic level, (2) presence of recalcitrant subpopulations of bacteria (known as persisters) that have become phenotypically drug tolerant in response to a variety of stresses ranging from drug exposure to immune pressure, nutrient shift, acidic pH, and low-oxygen tension (Sarathy et al., 2018). Persisters are defined by a “quiescent (non-growing or slow-growing) subpopulation of organisms that survive exposure to a bactericidal antibiotic, are genetically indistinct from drug-susceptible bacteria, and have the ability to revive under antibiotic-free conditions” (Mandal et al., 2019). Persisters are associated with reduced metabolic rate, activated stress response, and altered cell-wall permeability as compared to drug-susceptible bacilli, and are primarily established in macrophages or granulomatous lesions inside the human host (Mandal et al., 2019). Besides being associated with LTBI, persisters are thought to contribute to the requirement for lengthy anti-TB treatment and to play a significant role in relapse (Mandal et al., 2019).

A multi-stress model of non-replication that mimics some of the microenvironmental conditions that *M. tuberculosis* encounters in the host has been put forward including acidic pH at 5.0, mild hypoxia (1% O_2_), flux of nitric oxide and other reactive nitrogen intermediates arising from nitrite at low pH, and low concentrations of butyrate (fatty acid) as carbon source to induce a non-replicating state (Gold et al., 2015). After exposure to chemical compounds for 3 days, bacterial survival is assessed by aerobic outgrowth for 7–10 days (Gold et al., 2015). It has been pointed out that a disadvantage of this type of model is the need for a recovery or outgrowth phase that implies bacilli being replicated, which makes interpretation more difficult (Early et al., 2019a). A rapid method (readout in 7 days) has been developed to measure bactericidal activity against non-replicating *M. tuberculosis*, which was induced at low pH (citrate buffer at pH 4.5), did not require the outgrowth period, and had an easily measured readout (measuring luminescence to detect viable *M. tuberculosis* strain constitutively expressing luciferase) with the minimum of manipulations (Early et al., 2019a). Compounds with bactericidal activity against non-replicating bacteria were identified employing a pH-sensitive green fluorescence protein screening approach devised to identify compounds that disrupt the ability of *M. tuberculosis* to maintain its internal pH in an acidic environment (Early et al., 2019b).

Models for *in vitro* replicating *M. tuberculosis* include a large number of aerobic assays using various microbiological media with differing carbon sources and read-outs to assess mycobacterial growth (Yuan and Sampson, 2018; Parish, 2020). The complexity of TB disease, however, implies that no single *in vitro* model can reliably predict *in vivo* efficacy (Parish, 2020). Accordingly, it is unclear and difficult to establish which models best represent the real metabolic state of, for instance, replicating and non-replicating *M. tuberculosis* in the various human host environments, let alone the effects of the metabolic states of infected patients (e.g., microbiome status, nutritional state, chronic diseases). Notwithstanding, it has been suggested that the use of infected macrophage screens appear most general and have the advantage of reflecting physiologic conditions to the greatest extent currently possible (Yuan and Sampson, 2018). At any rate, no screening model can replace the requirements of extensive follow-on experiments in the human host to ascertain efficacy, pharmacokinetics, pharmacodynamics, toxicity (e.g., specificity) and the mechanism of action to guide medicinal chemistry efforts when optimization is needed.

### The Long and Eventful Journey of Anti-TB Agents to Reach Targets

The path that an antitubercular drug candidate must take is long and plenty of obstacles, which often make the compound unable to reach its target. In short, an anti-TB agent designed to bind a specific drug target in *M. tuberculosis* must be “transported from the blood compartment to non-vascularized pulmonary lesions, diffuse into necrotic foci and the caseum, and permeate the lipid-rich cell envelope” of bacilli at concentrations that alter the function of its target and act upon for a time frame needed for killing of bacilli (Dartois, 2014). Moreover, orally administered drugs should also overcome a number of barriers imposed by the human host, including be stable and soluble at the acidic pH of the stomach, have good permeability through the small intestine, withstand the first-pass metabolism, show adequate permeability in lung and uptake into *M. tuberculosis* to reach the intracellular target(s) (Dartois, 2014). Furthermore, chemical stability under different physiological conditions of the multicellular structures that are characteristics of the TB pathology, such as necrotizing or caseum granulomas must be considered (Dartois and Barry, 2013; Sarathy et al., 2018). Taking these into consideration, ideally, new chemical compounds to treat TB should exhibit the following attributes (Wellington and Hung, 2018; Yuan and Sampson, 2018): (1) have favorable pharmacokinetic and pharmacodynamics profiles, which would imply good absorption and distribution, low host metabolism (slow clearance, high exposure, and high bioavailability), liver and kidney elimination rates that do not preclude reaching concentrations needed to produce human efficacy, and low toxicity to widen the therapeutic index, (2) not be a substrate for efflux pumps, (3) be chemically stable to increase shelf-life and avoid cold chain needed to reach remote regions with limited resources, (4) have appropriate physicochemical properties, including absence of polymorphs to have, for instance, predictable dissolution rates, (5) have low cost of synthesis, (6) good solubility to, for instance, not require costly formulation, (7) kill MDR- and XDR-TB strains, which would likely imply a new mode of action, (8) shorten treatment time by being effective against non-replicating bacilli and killing the diverse physiological states of in *M. tuberculosis*–infected human tissues, (9) be compatible with current antitubercular and HIV therapeutics as TB/HIV co-infection is prevalent, (10) and, ultimately, achieve a stable and relapse-free cure of TB.

Different strategies can be used to identify and develop new anti-TB candidates. The classic approach known as “drug-to-target” has contributed to the establishment of current therapies, while the so-called “target-to-drug” approach still encountered some difficulty in proposing a new anti-TB drug, especially in relation to translating results *in silico* and *in vitro* to contexts involving whole cells or organisms. Recently, these two strategies have been merged by researchers, with the intention of further increasing the chances of discovering new drug candidates for TB.

## Phenotypic Screens or Targeted-Based Approaches

**Figure 1:**
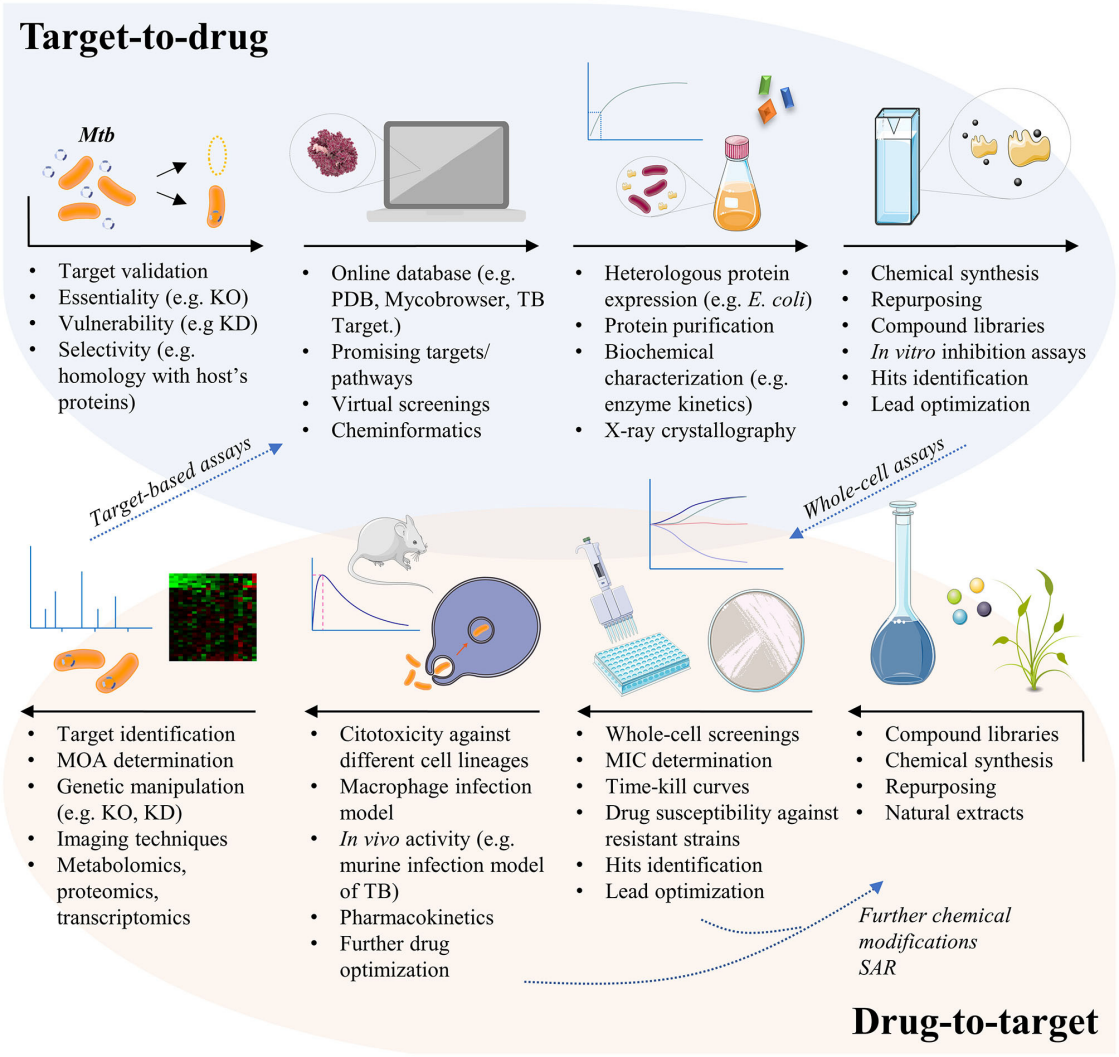
The different pathways for TB drug development. The target-based approach usually starts from genetic manipulation of *M. tuberculosis* cells to find essential, vulnerable, and selective molecular targets. Bioinformatics tools help researchers to test thousands of compounds against the given target by virtual screening, in order to select potential ligands *in silico*. Targets must be expressed, usually by heterologous expression in *Escherichia coli* cells, so that *in vitro* inhibition assays may be conducted. Finally, potential inhibitors of the given target must be tested in whole-cell assays to evaluate their activity against the mycobacteria and in different contexts of infection. The drug-to-target approach, also known as phenotypic screening, starts from compound libraries, natural extracts, or repurposing of known drugs, which are tested against the mycobacteria for minimum inhibitory concentration (MIC) determination. Active compounds may be also tested against resistant strains of *M. tuberculosis*, or in time-kill curves to have their mechanism of killing understood. Compounds may suffer additional chemical modifications that could enhance their antimycobacterial activity or to reduce potential cytotoxic effects, before been tested in other contexts of infection. The unknown target of the new lead must then be revealed, a challenging quest that is helped by a set of different methods. Finally, validated targets with already known inhibitors may follow the target-based pathway, in order to understand the inhibition mechanism and to identify the inhibitor binding site, for instance. KO, knockout; KD, knockdown; MOA, mechanism of action; PDB, protein data bank; SAR, structure-activity relationship; TB, tuberculosis.

At present, the initial screening for the development of a new drug follows a directed or empirical approach (Zheng et al., 2013). In the directed approach, the screening strategy is based on a specific target, i.e., the effect of the compounds against a specific enzyme is analyzed *in vitro*. In contrast, the strategy of the empirical approach is based on phenotypic screening, in which the effects or phenotypes induced by compounds in cells, tissues, or whole organisms are evaluated (Swinney, 2013a,b). Both approaches (Figure 1) have advantages and disadvantages for drug development efforts.

### Target-Based Approach

Target-based approaches are pivotal for guiding chemical lead optimization and in toxicology studies during preclinical development. However, it has been recognized that target-based drug discovery may have some limitations in clinical trials due to the poor correlation of models with human diseases (Zheng et al., 2013). Target-based screenings for antibacterial and antifungal drug discovery have yielded disappointing outcomes due to a few reasons such as: (1) compounds active on purified enzyme target (*in vitro*) did not always enter screens; (2) many lead compounds identified were non-specific inhibitors; (3) compounds were not always adequately orally bioavailable (Goldman, 2013). Moreover, obtaining molecules with cell permeability without cytotoxicity through medicinal chemistry can be a very slow and difficult process (Payne et al., 2007). At any rate, the following advantages of the target-based approach to the development of new chemical entities (NCEs) against TB may be taken into consideration: (1) the search and identification of lead compounds with defined molecular mechanisms against a defined target (e.g., enzymes from defined pathways), (2) the analysis of compounds with a favorable cost/benefit ratio, (3) the development of compounds with selective toxicity (the fundamental principle of chemotherapy), even in the initial stages, (4) structural data of target, when available, help guide medicinal chemistry efforts to improve pharmacodynamics and pharmacokinetics, (5) the pre-clinical evaluation of lead compounds, (6) and generation of patents if a new mode of action is unveiled or a new chemical scaffold is proposed. Moreover, phenotypic screening efforts may be followed by identification of target(s) and, if amenable, heterologous expression, purification, protein assay, and structural determination to help guide medicinal chemistry efforts can be pursued (closing full circle) (Figure 1).

### Phenotypic Screening Approach

Usually, hit candidates for infectious diseases come from screens that involve intact pathogens, akin to phenotypic screens (or high-content screens) rather than target-based screens (Katsuno et al., 2015). Interest in whole-cell and target-based whole-cell screening campaigns have thus been rekindled due to, at least, two reasons: (1) current TB drugs were discovered in whole-cell screens for inhibition of *M. tuberculosis* growth or growth of a surrogate of *M. tuberculosis*; (2) even though the availability of whole genome sequence of *M. tuberculosis* prompted the search for novel targets for anti-TB agents, target-based screens have not yielded a successful anti-TB agent (Yuan and Sampson, 2018). Target-based enzymatic assays strive to detect *in vitro* inhibition of homogenous proteins and are generally driven by improved binding affinity (that would result in better target specificity and lower off-target binding probability). However, these assays neglect pivotal properties, such as cell wall permeability, metabolic stability, and drug target vulnerability (Yuan and Sampson, 2018).

A review of the first-in-class small molecule drugs approved by the FDA between 1999 and 2008 revealed that empirical phenotypic screening had much more success for the discovery of new molecular entities than target-based approaches (Swinney and Anthony, 2011). Therefore, several companies (e.g., Novartis AG and GlaxoSmithKline) and research centers have focused on phenotypic screening as a significant tool for drug discovery (Ballell et al., 2005; Payne et al., 2007). This phenotypic approach associated with the screening of the inhibition of cell growth has become a more rapid and efficient strategy for identifying first-in-class small molecules (Swinney and Anthony, 2011). This random approach has found some success with clinical-stage antimycobacterial drugs, such as nitroimidazoles (pretomanid and delamanid) (Stover et al., 2000), 1,2-diamine SQ-109 (Lee et al., 2003), and bedaquiline (Andries et al., 2005). Incidentally, in August 2019, the TB Alliance received approval from the U.S. Food and Drug Administration (FDA) for pretomanid for the treatment of adults with pulmonary extensively drug-resistant tuberculosis (XDR-TB) and multidrug-resistant TB (MDR-TB) that is treatment-intolerant or non-responsive (www.tballiance.org). Pretomanid was approved as part of a three-drug, 6-month, all-oral combination regimen with bedaquiline and linezolid (collectively referred to as the BPaL regimen) (Grüber, 2020).

As pointed out in 2.2 above, current *in vitro* models relevant to host infection employed in anti-TB discovery campaigns may include replicating bacteria (aerobic growth, carbon source variation), non-replicating bacteria (nutrient starvation, low oxygen states, lipid-rich environments, low pH models, streptomycin-dependent strains, multi-stress models), persistence and antibiotic tolerance, intracellular bacteria (macrophage cell lines, primary cells), complex host-pathogen interaction models (caseum, granuloma), and zebrafish larvae model using a fluorescent strain of *Mycobacterium marinum* (Parish, 2020). As a compreensive review on the various types of phenotypic screening approaches is beyond the scope of our contribution, the readers are referred to excellent reviews that address these experimental strategies such as those given by Wellington and Hung (2018); Parish (2020), and Yuan and Sampson (2018).

At any rate, whether target-based approach or phenotypic screening is more productive is debatable. For instance, the high cost and uncertainty that are inherent to the phenotypic drug discovery process limit hit progression (Comess et al., 2018), whereas the drawbacks of target-based approach are mentioned in section Target-Based Approach above.

## Desirable Features of Targets and Strategies for Validating Novel Targets

The complete genome sequencing *of M. tuberculosis* H37Rv strain has accelerated the study and validation of molecular targets aiming at the rational design of anti-TB drugs (Cole et al., 1998). The complete genome sequencing has provided impetus for the advancement of experimental strategies including, but not limited to, targeted genetic manipulation, transcriptomics, metabolomics, proteomics, structural genomics, and comparative genomics, which, in turn, was facilitated by whole genome sequencing (Lechartier et al., 2014). A promising target must be involved in a fundamental process for the survival or virulence of the pathogen and should, preferentially, be absent from the human host to hopefully result in the development of non-toxic therapeutic agents to treat TB. Alternatively, a promising target may play a key role in the bacilli metabolism in latent and/or persistence states. Besides being essential *in vivo*, an ideal target for antibiotic development should also be drug vulnerable and druggable.

### Target Essentiality

A typical first step to establish essentiality of a gene product is genome manipulation of the bacilli, usually by knocking out the target gene function. Essentiality of a potential drug target may be evaluated by genetic and chemical approaches (Sassetti et al., 2003; Wei and Rubin, 2008; Rancati et al., 2018), and its first evidence generally arises from negative data—the inability to rescue viable bacilli in the absence of a given gene. Several methods are now available for researchers to generate knockout mutants. Chhotaray et al. (2018) and Borgers et al. (2019) have reviewed the mycobacterial genetic toolbox and proposed a guide for generating mutants in slow-growing mycobacteria from the *M. tuberculosis* complex. Some methods are useful for site-directed gene deletion or replacement, including allelic exchange using mycobacterial plasmids (Snapper et al., 1990; Pelicic et al., 1997; Parikh et al., 2013), specialized transduction (Bardarov et al., 2002), double- and single-stranded recombineering (van Kessel and Hatfull, 2007, 2008), enhanced specialized transduction using recombineering (Tufariello et al., 2014), and most recently the ORBIT system (Murphy et al., 2018). Others are more suitable as screening tools or for creating large mutant libraries, such as the transposon random mutagenesis (Griffin et al., 2011; DeJesus et al., 2017). Each genetic tool carries its own methodological difficulties. Although methods based on shuttle plasmids may be time consuming, as several cloning and selection steps before isolating a single mutant are needed, they offer the possibility of creating specific and “scarless” gene knockouts. Bulk creation of mutant libraries by transposon mutagenesis has the advantage of generating thousands of mutants in a few steps, however isolation of these mutants for further validation experiments and creation of clean knockout strains to avoid polar effects are cumbersome (Borgers et al., 2019). Notwithstanding, most gene essentiality data currently available for *M. tuberculosis* are derived from saturating transposon mutagenesis, and a good starting point for investigating a potential target is to access these data at TB databases, such as Mycobrowser (http://mycobrowser.epfl.ch/) (Kapopoulou et al., 2011) and the TB TARGET programme (http://webhost.nts.jhu.edu/target) (Chhotaray et al., 2018). The need for a biosafety level 3 facility for the genetic manipulation of *M. tuberculosis*, which imposes a considerable barrier to be overcome by many TB research groups to develop their projects, is being circumvented by the generation of biosafety level 2 avirulent strains, such as the *M. tuberculosis* MC^2^6230 (ΔpanCD ΔRD1) (Sambandamurthy et al., 2002; McNeil and Cook, 2019).

The cutting-edge ORBIT (Oligonucleotide-mediated Recombineering followed by Bxb1 Integrase Targeting) technology has also contributed to lower the obstacles met by genetic TB researchers. This system consists of a combination of two phage recombination systems that enables deletions, insertions, promoter replacements and other chromosomal engineering in *M. smegmatis* and *M. tuberculosis* (Murphy et al., 2018). Two phage proteins are involved in this system, the Che9c RecT annealase and the Bxb1 integrase, and the host cell that contains a plasmid expressing these proteins. The first step is the coelectroporation of a single-stranded oligonucleotide and a non-replicating “payload plasmid.” The “targeting oligo” carries the Bxb1 *attP* site internally and anneals at a specific sequence in the lagging strand template of the target gene, helped by the RecT enzyme. The “payload plasmid” contains the corresponding Bxb1 *attB* recombination site, a hygromycin resistance gene as a selectable marker, and additional tagging sequences (e.g., green fluorescence protein coding sequence). Finally, the *attP*-*attB* site-specific recombination is mediated by the Bxb1 integrase, resulting in the integration of the “payload plasmid,” which can be cured for plasmid-free strains if desired. ORBIT proved to be a very efficient technique, in which 20–200 clones are typically obtained and only 2–4 candidates need to be analyzed to detect the desirable clone. The authors reported the knockout of more than 100 genes and proposed that the ORBIT technology may also be employed in studies of essentiality and vulnerability of gene products, elucidation of putative genes' function, drug resistance, construction of mutant libraries, among others (Murphy et al., 2018).

Regardless of the genetic tool that a researcher may choose to investigate the essentiality of a given gene, the context in which the new mutant strain will be evaluated is of utmost importance. Genes that are non-essential under ideal *in vitro* growth conditions could play an essential role in bacilli infection and/or survival inside the host, persistence, or latency. Hence, essentiality should be taken as a relative concept, in the sense that it may vary according to the environment in which a mutant strain is growing (Rancati et al., 2018). Mycobacterial genes that are shown to be essential under *in vitro* growth conditions are usually expected to be fundamental for the *in vivo* infection context also. However, context-dependent roles have been shown, for instance, for Ami1 (N-acetylmuramyl-L-alanine amidase; Rv3717) and RipA (D,L-endopeptidase; Rv1477) enzymes, which are involved in synthesis and cleavage of mycobacterial peptidoglycan polymer (Healy et al., 2020). Ami1 was shown to be dispensable for cell division of *M. tuberculosis* in different growth contexts *in vitro* (e.g., macrophage infection, nutrient starvation, and nitric oxide exposure), but it was critical for persistence in the lungs of infected mice (Healy et al., 2020). RipA impaired cell division in culture and was shown to be important for growth inside macrophages *ex vivo*, and for cell survival inside the host (Healy et al., 2020). These authors have thus suggested that D,L-endopeptidase RipA could represent a target for the design of new inhibitors as its depletion halts replication within macrophages and leads to clearance of *M. tuberculosis* from infected mice. The transcriptional factor MpbR of *M. tuberculosis* was described as important for the biosynthetic regulation of cell-wall lipids, colony morphology and biofilm formation, and the absence of its activity in the context of infection of murine hosts led to reduced lung bacterial burden and pulmonary inflammation (Li et al., 2019). The ESX-1 or type VII secretion system comprises ~20 genes that releases effectors into the extracellular milieu (Sala et al., 2018). The EspL protein is a component of the EDX-1 secretion system and the Δ*espL* deletion mutant did not show any major difference as compared to the wild type strain during *in vitro* growth in standard medium (Sala et al., 2018). By contrast, EspL was shown to be essential for mycobacterial replication within the macrophages, to elicit host production of cytokines and to manage the production of other components of the secretion system (Sala et al., 2018). Evaluation of phenotypes resulting from gene knockout experiments should, therefore, be carried out under various conditions, both *in vitro* and *in vivo*, to assess the essentiality of a gene product in different environmental contexts and different replication states that are relevant for the infection establishment (Hingley-Wilson et al., 2003).

### Target Vulnerability

It might be expected that chemical inhibition of a genetically essential gene product would lead to the same phenotype as observed in the gene knockout strain (Yuan and Sampson, 2018). However, the vulnerability of target should also be evaluated as it plays an important role. Vulnerability is related to the degree of target inhibition needed to affect cell viability, which, in turn, leads to bactericidal drug effect. Low vulnerability of an essential gene product may require 100% occupation of sites of the intracellular protein target (total target engagement), and this phenomenon had already been described for some mycobacterial targets (Reddy et al., 2014; Park et al., 2017). Achieving almost 100% of inhibition of target function is unlikely and, thereby, difficult to be drugged as the physiologic change will not be brought about. Reduction of expression level of the target protein (gene knockdown) may be employed to evaluate vulnerability (Evans and Mizrahi, 2015). Conditional knockdown mutants (“hypomorphs”) in which expression of the target gene is switched on upon addition of anhydrotetracycline (ATc) are referred to as “Tet-ON” mutants (Evans and Mizrahi, 2015). In short, in the absence of tetracycline (Tet), Tet repressor (TetR) dimers bind to upstream Tet operators (*tetO*) thereby repressing transcription of *tetA*, Tet-exporting protein coding sequence. Addition of ATc and its subsequent binding to TetR triggers a conformational change that results in dissociation of TetR from *tetO*, enabling Tet-mediated transcription of *tetA*, which in turn transports Tet across the cytoplasmic membrane to the periplasm of Gram-negative bacteria (Evans and Mizrahi, 2015). Removal of the ATc inducer silences target gene expression of Tet-ON mutants. Dose-dependent regulation of genes may be brought about by expression of Tet repressor (TetR) from either a strong promoter or an intermediate-strength promoter to generate hypomorphs in, respectively, the Tet-ON_S_ and Tet-ON_M_ configurations (Evans and Mizrahi, 2015). As the removal of ATc can be difficult to achieve in some experimental settings, the manipulation of Tet-dependent hypomorphs was simplified by the development of a modified “Tet-OFF” system, which utilizes a mutated, “reverse” TetR (revTetR) that binds to Tet operators (*tetO*) only in the presence of ATc (Evans and Mizrahi, 2015). This system enables the generation of mutants in which target gene expression is repressed upon addition of the ATc effector. The regulatory capacity of both Tet-ON and Tet-OFF systems in mycobacteria has been enhanced by adapting the codon usage of the genes encoding TetR and revTetR to *M. tuberculosis* genome, allowing increased expression in mycobacteria (Klotzsche et al., 2009). Knockdown can be achieved by engineering expression of antisense RNA to modulate mRNA levels or by replacing native promoter, as described above, with a controlled one that transcribes the target gene in the presence or absence of inducer (e.g., ATc) allowing titration of protein target (Wei and Rubin, 2008).

A CRISPR-Cas9 (clustered regularly interspersed short palindromic repeats-CRISPR associated proteins) system has been repurposed (CRISPR interference or CRISPRi) to allow transcriptional repression in mycobacteria (Rock et al., 2017). This optimized CRISPRi platform for mycobacteria appears to be the simplest and fastest method for programmable gene regulation as pointed out by these authors, and works using this tool as a drug validation strategy are starting to emerge (Landeta et al., 2019; McNeil and Cook, 2019). The CRISPRi tool has been merged with Tn-Seq analysis to create a new method (CRISPRi-Seq) that can identify and characterize essential mycobacterial genes (de Wet et al., 2018). These authors created a pooled CRISPRi library targeting 2,385 *M. smegmatis* homologs of *M. tuberculosis* genes, and employed *in silico* designed 11,467 sgRNAs with up to five sgRNAs per gene cloned into the delivery dCas9-expressing vector. Although CRISPRi-Seq uses conditional gene knockdown instead of gene knockouts or transposon mutagenesis, which are usually used for assessing gene essentiality, it could identify 80% of genes that had been previously classified as essential by Tn-Seq in *M. smegmatis* (de Wet et al., 2018), and is now part of the repertoire of genetic techniques to identify new essential targets. The CRISPRi tool has also been combined with image-based analyses and assessment of morphological outcomes of 272 essential gene knockdown mutants of *M. smegmatis*, an approach that also promises to help elucidation of the physiological role of genes with unknown or *in silico* predicted function (de Wet et al., 2020). The vulnerability of a functional target can be evaluated by controlling protein levels using inducible protein degradation rather than regulating mRNA levels allowing studies of long-lived proteins in native expression levels (Wei et al., 2011). This approach employs the conserved Clp protease system consisting of highly processive ClpP protease and accessory proteins (ClpX, SspB) that identify C-terminus SsrA-tagged proteins and help denature them before proteolysis (Wei et al., 2011). A dual control switch system has been engineered in which a single inducer—ATc or doxycycline (doxy)—simultaneously triggers transcriptional repression of a target gene and degradation of the encoded protein target (Kim et al., 2013). This dual control system has been employed to identify and evaluate gene vulnerability of proteins required for growth and non-replicating persistence *in vitro* and during infections (Kim et al., 2013). Cell washout experiments may be used to assess target vulnerability for drugs with long residence times (slow off rate constants) (Walkup et al., 2015). Target vulnerability should be evaluated under different *in vitro* and *in vivo* conditions as target expression may depend on the environmental milieu. Some drug targets can exist at levels much higher than are needed to support growth (Wei et al., 2011). For instance, a larger than 97% depletion of dihydrofolate reductase (DHFR) and alanine racemase (Alr) only modestly slowed growth, whereas modest depletion of RNA polymerase β-subunit (RpoB) and efficient depletion of enoyl reductase (InhA) led to growth cessation suggesting that the latter two biochemical processes are hypersensitive to inhibition (Wei et al., 2011).

### Target Druggability

It has been estimated that 60% of small-molecule drug-discovery projects fail because the target is found to not be druggable (Halgren, 2009), implying that target function will not be affected by the drug candidate. “Undruggable” (not druggable) targets are unlikely to be affected by a drug candidate. Halgren (2009) has listed the characteristics of protein sites to assess “druggability” of targets. “Undruggable” (not druggable): (a) “very strongly hydrophilic; relatively small in size; little or no hydrophobic character; or (b) requires covalent binding; or (c) very small or very shallow (extensively exposed to solvent).” “Difficult:” “sufficiently hydrophobic to require administration as a prodrug to protect charged chemical groups (ligands with charged chemical functions may impair passive transport); less hydrophobic than a typical site.” “Druggable” (target can be reached and influenced by a particular chemical compound): “of reasonable size, enclosure (not extensively exposed to solvent), and hydrophobicity with unexceptional hydrophilicity (sites with increased polarity are less likely to be druggable).” The target site size is an important indicator of druggability as “the larger the better opportunities to make chemical modifications to optimize physicochemical properties (e.g., solubility) without affecting ligand affinity to protein target” (Halgren, 2009). Computational subtractive genomics approach has recently been employed to identify hypothetical proteins as druggable targets in XDR-TB strains (Uddin et al., 2018). Notwithstanding, whether or not this approach will bear fruit remains to be shown. Mutation in the *atpE* DNA sequence, encoding the *c* subunit of ATP synthase, detected by whole genome sequencing of drug-resistant isolates and complementation studies identified mycobacterial ATP synthase as the drug target of bedaquiline (Andries et al., 2005). As pointed out by Lechartier et al. (2014), these findings demonstrated the energy metabolism and inhibition of ATP synthase in particular as highly druggable targets.

Finally, it is desirable that a three-dimensional structure of a protein target be available to help guide medicinal chemistry efforts (Gashaw et al., 2011). In the case of enzymes as targets, availability of a direct and continuous method for *in vitro* biological activity determination (assailability) would enable high throughput screening (HTS) efforts (Gashaw et al., 2011). For drug candidates that are inhibitors of enzyme activity a deep understanding of the enzyme, chemical, and catalytic mechanisms are needed for medicinal chemists to devise changes aiming at decreasing the overall dissociation constant of ligand-protein interaction. Determination of the mode of action of an enzyme-catalyzed chemical reaction can aid in the design of screening efforts (Holdgate et al., 2018). Moreover, studies of the type of inhibition of a chemical compound are needed as there are preferrable modes of action for hits to justify chemical optimization efforts to be pursued (Holdgate et al., 2018). For instance, the weakness in the use of classical competitive enzyme inhibitors as drugs for clinical conditions is that inhibition results in the upstream accumulation of the substrate for the enzyme, which may overcome the inhibitior effect and ensuing increase in enzyme activity (abrogating the desired physiological effect).

## Function, Thermodynamics, Structure, Bioinformatics, cheminformatics, and Medicinal Chemistry Bearings on Drug Discovery

**Figure 2:**
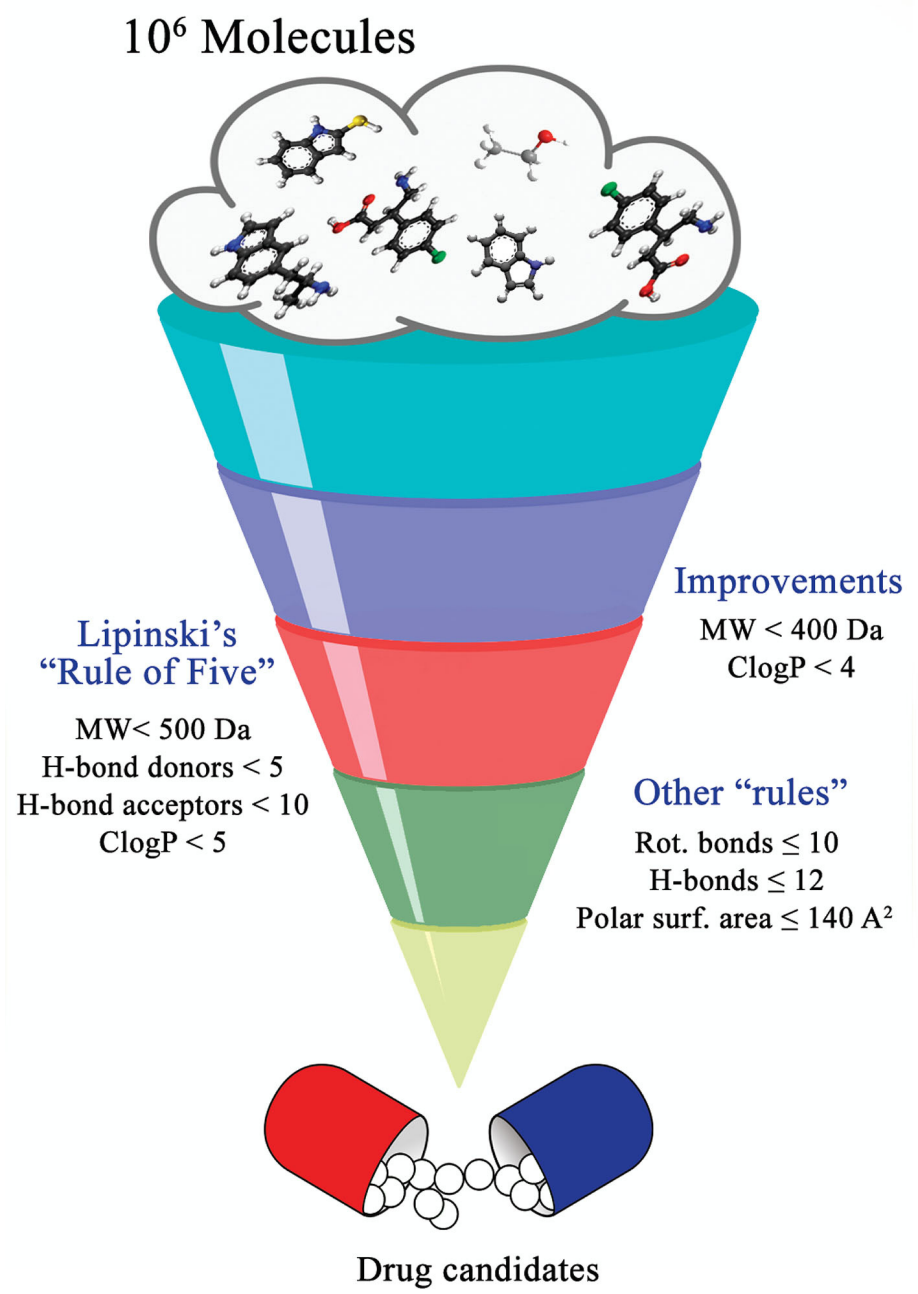
Guiding rules for chemical compound screening.

**Figure 3:**
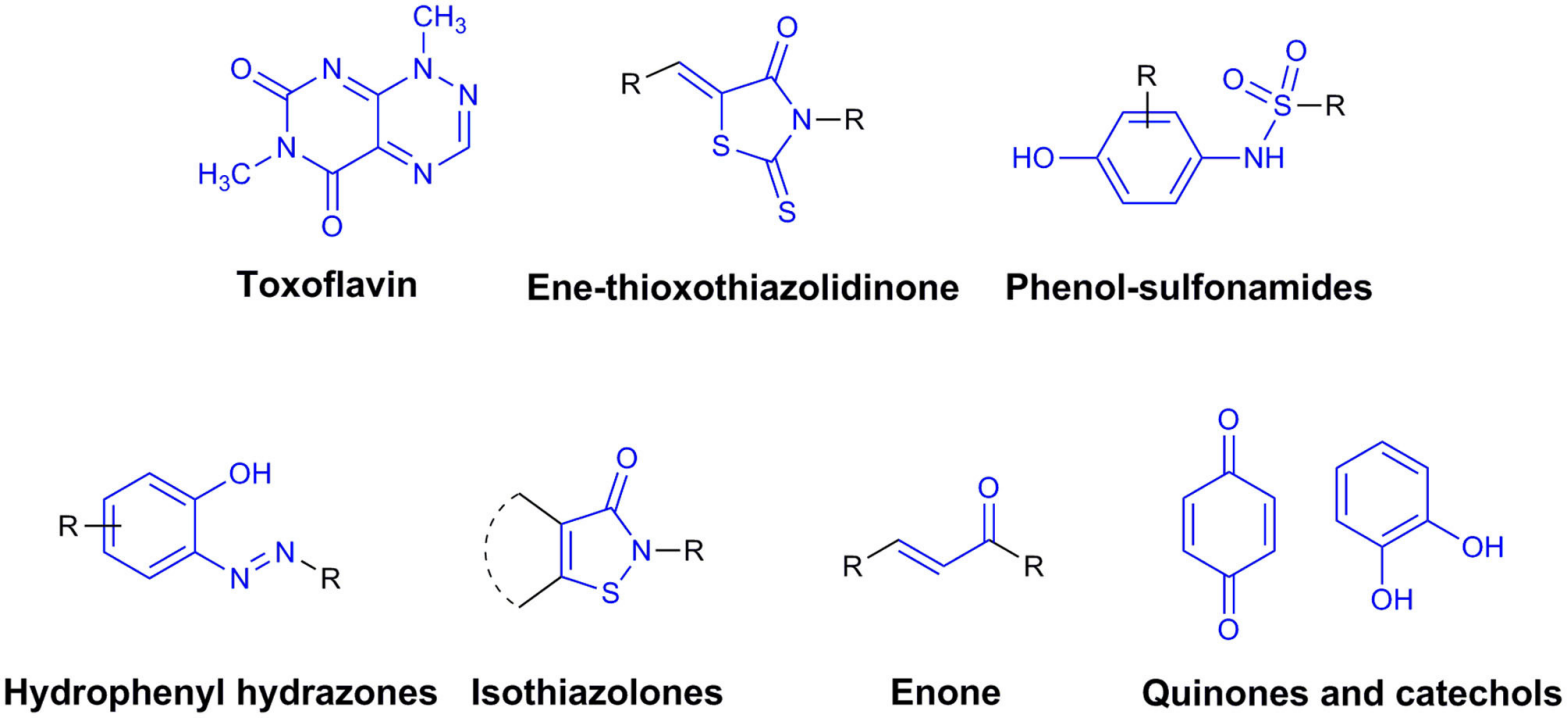
Pan-Assay Interference Compounds (PAINs).

By definition, drug discovery implies exploration of unknown. Notwithstanding, the discovery process may be influenced by what is known about a target, a phenotypic outcome or a specific and desirable chemical compound profile. These, in turn, represent biases that may exert leverage on the choices of outcomes that are considered as successes when selecting the first set of chemical compounds to be screened. The “rule of five” put forward by Lipinski (Lipinski et al., 1997; Lipinski, 2016) based on the physicochemical profiles of phase II drugs and the rules put forward by Veber (Veber et al., 2002) for increased oral bioavailability in rats (Figure 2) serve as guidelines in the development of new drugs and/or lead optimization. Gleeson has suggested that ADMET characteristics are improved when MW <400 Da and CLogP <4 (Gleeson, 2008). However, it is usually difficult to predict whether a chemical modification and/or addition/removal of a functional group of a hit compound will result in better activity. Normally the immune system does not respond to xenobiotics with MW < , 1000 Da (Copeland, 2013), which could be considered a further filter for drug development. In addition, some chemical classes of compounds that appear to be hits and are truly artifacts for drug development have been named “Pan-Assay Interference Compounds” (PAINS) (Figure 3) and a few examples of them have been fully analyzed by Baell and Walters (Baell and Walters, 2014). Interestingly, it has recently been reported that anti-TB agents do challenge established rules as they are more lipophilic than many other antibiotics (Machado et al., 2018). This may be due to the waxy mycobacterial cell wall that reduces internalization of more hydrophilic compounds (Machado et al., 2018). For instance, Bedaquiline has a ClogP value of 7.10 and Clofazimine a ClogP value of 8.43, which are anti-TB candidates in Phase 3 clinical trials. Further examples may be found for chemical compounds in Phase II clinical trials (ClogP values of 5.09 and 5.82 for, respectively, PBTZ-169 and SQ-109) and Phase I clinical trials (ClogP values of 7.49 and 7.10 for, respectively, TBI-166 and Q-203) (Machado et al., 2018).

Effectively, the possibilities of chemical derivatization from a lead structure are fairly numerous, and many potential analogs can be envisaged. It has been estimated that the chemical space of small molecules with molecular weight of 500 or less and containing the common atoms found in drugs is estimated at 10^60^ (Dobson, 2004). Because of the impossibility of preparing all of the imagined compounds, according to Wermuth (2008), some classical rules should be applied to increase priority selection in time- and financial resource-saving manners such as follows:

1. *Minor modification rule*: modest modifications in the leading compounds, through very simple organic reactions (e.g., methylation, reduction, hydrogenation, acetylation). This strategy permits the solid exploration of the function of each chemical group to determine their physicochemical influence on activity.
2. *Biological logic*: some chemical groups are prone to yield toxic metabolites, such as nitroso, azo, amino, hydrazines, and hydroxylamines, and should be avoided. In contrast, the addition of chemical groups can modulate the metabolic profile of lead compounds that favor or slow down biodegradation (e.g., insertion of halogens).
3. *Structural logic*: taking into account all of the available structural data through comparison of known active compounds, in an attempt to derive important physicochemical features associated with potency, selectivity, and pharmacokinetic profiles. Some researchers refer to this method as pharmacophore identification.
4. *Correct substituent choice*: its main objective is to minimize the number of test compounds that have to be synthesized to ensure a significant steric, electronic, and lipophilic space. Within this context, the thermodynamic Craig plot [of δΔH vs. δ(–TΔS)] for each agent (Baggio et al., 2018) and the decision tree of Topliss (Jorge et al., 2011) allows the identification of moieties associated with efficacy.
5. *Eliminate or reduce chiral centers*: it has been described that racemates and enantiomers are three different pharmacological entities and that each one requires extensive pharmacological characterization. From the pharmacological and synthetic points of view, asymmetry-induced synthesis is not a trivial process in the pharmaceutical industry context.

Taking these rules into consideration, the optimization of lead-like compounds either from natural sources or from high-throughput screening (HTS) campaigns would be accomplished and hopefully result in novel compounds with antitubercular activity. Additionally, cheminformatics methods have been adopted as a complement to the traditional ones. These computational methods manage, mine and/or simulate complex systems or processes, whether they are chemical, genomic, proteomic, or clinical data (Ekins et al., 2011). This way, the integration of different methods ought to be useful for compounds selection for *in vitro* screens, and for the identification of new compounds as antitubercular hits or leads.

**Figure 4:**
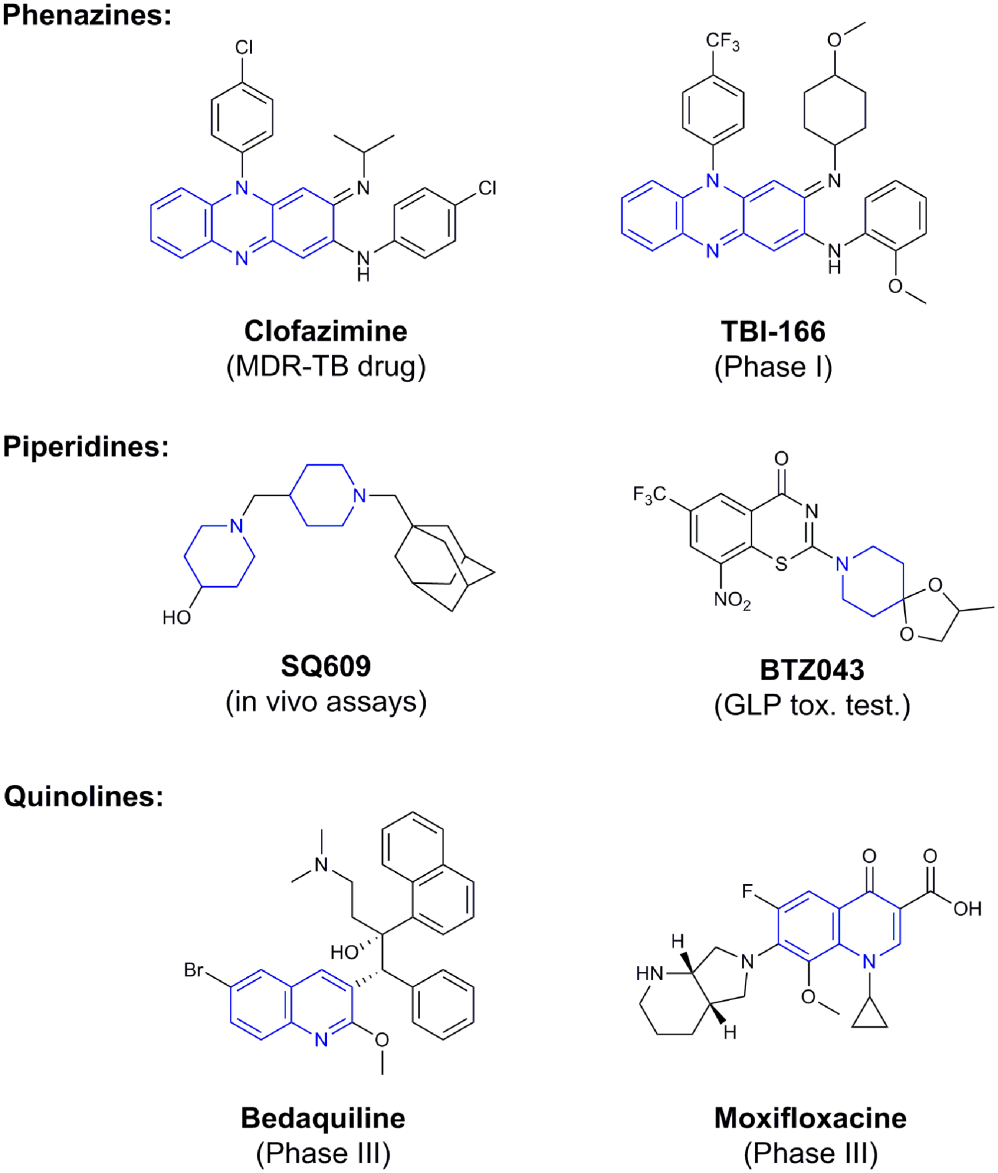
Valuable scaffolds and drugs obtained from natural sources.

Phenotypic assays are important for obtaining novel compounds with pharmacological activity (Swinney and Anthony, 2011). Subsequently, the target(s) could be discovered through chemical biology, genomics and proteomics strategies. However, even in the absence of a known molecular target, medicinal chemistry efforts can improve the efficacy of drug candidates by modifying specific parts of the molecule and evaluating their effect on activity (Bleicher et al., 2003). A strategy using an *a priori* disjunctive approach by molecular simplification may be employed to identify lead structures from natural sources, which generally show a high degree of structural complexity. This molecular dissection reduces the molecular complexity by deleting functions and molecular elements that do not form part of the pharmacophore lead—a molecular simplification strategy. This method can lead to the preparation of molecules with facilitated synthetic access, thereby allowing scaling up of compounds for subsequent assays (Quan et al., 2017). By using this strategy, phenazines, piperidines, and quinolines have been identified as valuables scaffolds for the development of new anti-TB drugs. Some of the screened/developed compounds are now in use or in late stages in drug development (Figure 4).

**Figure 5:**
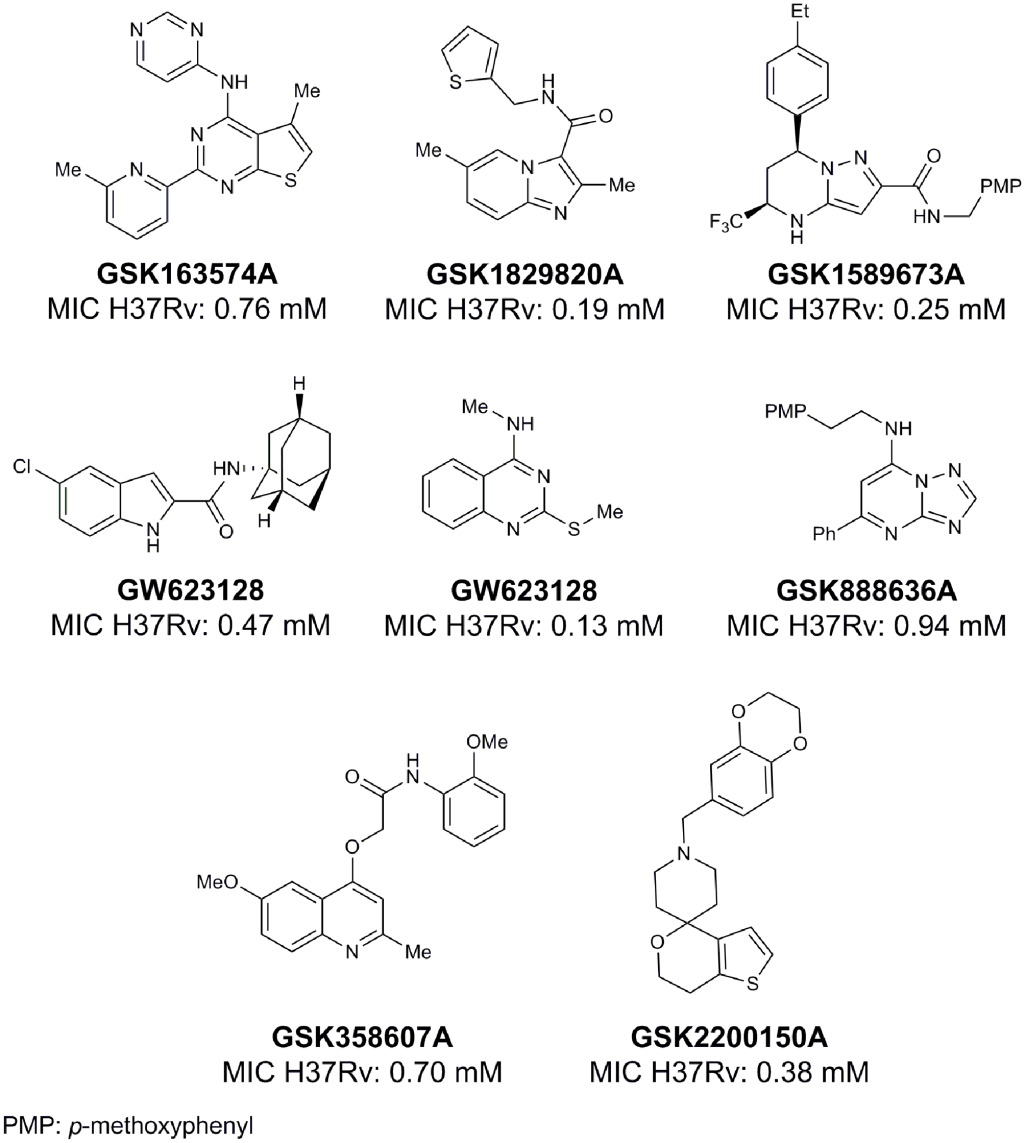
Leading compounds obtained through open source high-throughput screening.

**Figure 6:**
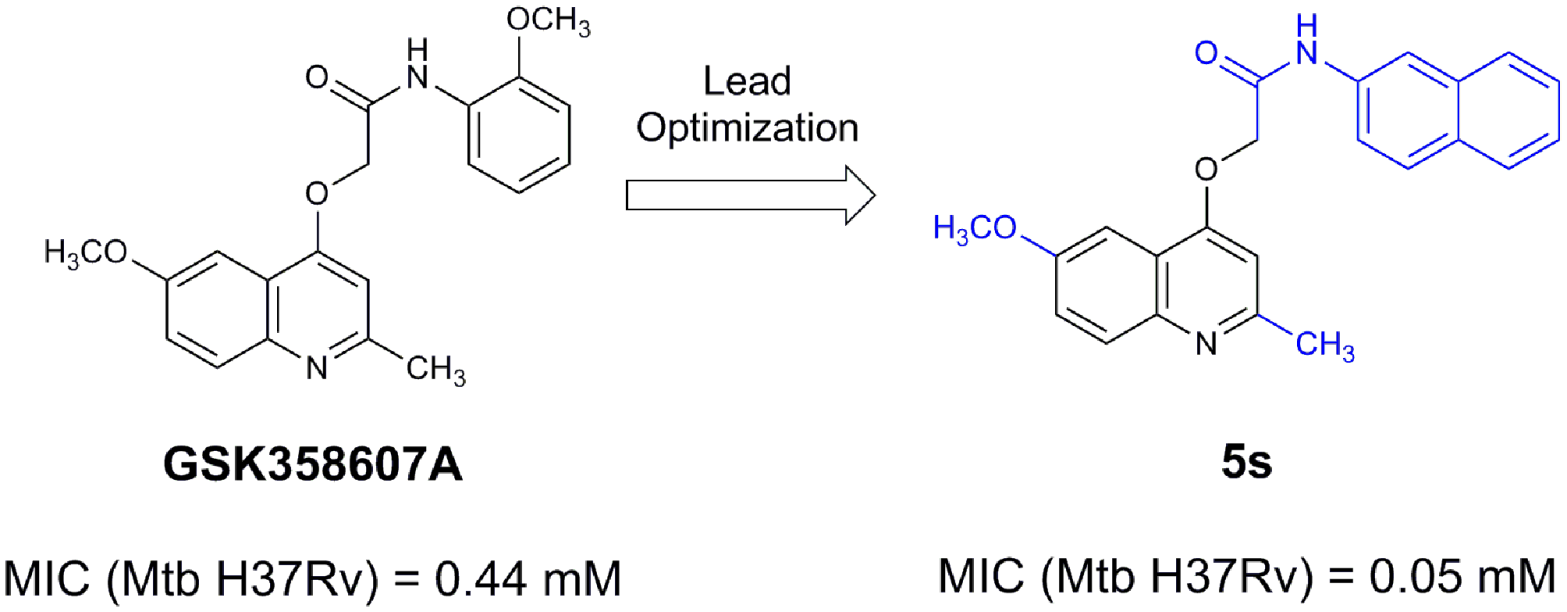
2-(Quinolin-4-yloxy)acetamides compounds actives against *M. tuberculosis*.

From lead-like compounds obtained through HTS campaigns, two approaches can be used to optimize structures: analogical and conjunctive strategies. In the analogical strategy, the degree of complexity of the candidate is maintained, and the modifications involve only isosteric and bioisosteric replacements (Lima and Barreiro, 2005) aiming at understanding the contribution of chemical groups to activity and achieving activity improvements. Using this approach, the establishment of electronic, steric, and lipophilic/hydrophilic gradients in the proposed modifications allows the identification of important structure-activity relationships (SAR). For example, Ballell and co-workers identified seven classes and one singleton with potential to be active against *M. tuberculosis* (Ballell et al., 2013). These compounds were identified out of ~20,000 compounds following a number of screens that evaluated their inhibition of mycobacterial growth, cytotoxicity, and physicochemical properties. The best compounds in terms of bioactivity are depicted in Figure 5. In the conjunctive strategy, attachment of additional chemical groups to the molecules is employed in an attempt to obtain better pharmacological profiles (efficacy, toxicity, and pharmacokinetic parameters). Moreover, the conjunctive approach can utilize the concepts of privileged chemical groups and structures for the construction of novel antimycobacterial agents. These molecular frameworks are able to provide ligands for diverse targets and have been used in medicinal chemistry programs for obtaining novel active compounds (DeSimone et al., 2004; González et al., 2012). For instance, 2-(quinolin-4-yloxy)acetamides (previously identified in Ballell's work) were investigated in a preliminary structure–activity relationship (SAR) study and have been described as potent *in vitro* inhibitors of *M. tuberculosis* growth (Pissinate et al., 2016), and, using a conjunctive strategy, chemical modifications of lead compounds yielded antimycobacterial agents with minimum inhibitory concentration (MIC) values as low as 0.05 μM (Figure 6). Quantitative Structure-Activity Relationships (QSAR) analysis allows the quantitative correlation of the characteristics of molecules with activity. For the construction of these models, the equilibrium constants and rate constants that are used in the proposed functional tests (e.g., enzyme activity measurements) for medicinal chemistry should be related to the free energy values ΔG. Therefore, only equilibrium and rate constants pass muster in terms of the free-energy relationships or influence on QSAR studies. The data are expressed on a logarithmic scale because of the linear relationship between the response and the log dose in the midregion of the log dose-response curve. The inverse logarithms of the activity (log(1/activity)) are used such that higher values are obtained for more effective analogs (Patrick, 2013). For instance, combining QSAR and molecular docking studies, respectively, predicted the anti-tuberculosis activity of 4-Alkoxy-Cinnamic derivatives and shed light on interactions between ligands and the target site of *M. tuberculosis* DNA gyrase (Adeniji et al., 2020).

When information of targets is available, efforts should be directed to obtaining novel and favorable intermolecular interactions between ligands and targets. The exploration of unoccupied cavities through the establishment of new interactions improves the stability of target-ligand complex, thereby leading to improved activity. Coupled with computer-assisted drug design (CADD), if X-ray 3D structural data are available, it is possible to match the different candidates or the lead. As a result, the distances between the chemical groups of the ligand and the molecular target can be studied culminating in the proposition of new structures with the aim of achieving better interactions (Freire, 2009). The stability of a biological complex is determined by the binding free energy (ΔG), which is comprised of the enthalpy (ΔH) and entropy (ΔS) terms (Fisher and Singh, 1995). The binding enthalpy represents the loss of non-covalent associations, such as hydrogen bonds with water molecules, and the subsequent formation of interactions between the ligand and macromolecule in addition to the reorganization of the displaced solvent molecules (Freire, 2004). The binding entropy term considers the displacement of solvent from the binding surfaces and the loss of conformational freedom (Murphy et al., 1994). In general, larger binding affinity values resulting from increased intermolecular interactions leads to enthalpic properties with more negative (favorable) values. Moreover, the interplay between the release of water upon binding (increased entropy) and the reduction of conformational freedom of the ligands (decreased entropy) should result in net positive entropy to produce compounds with higher affinity. Another approach to reduce the free energy of the system under study is to accomplish conformational restriction of ligands, which would reduce the loss of degrees of freedom upon binding, and thus reduce the entropy penalty to yield compounds with higher affinity. Similarly, the improvement of the lead lipophilicity increases entropy by promoting the release of solvation water molecules upon binding to the target. Therefore, more lipophilic compounds become alternatives for improving the antimycobacterial activity and thereby enabling optimized interaction with the proposed targets. Lipophilicity plays an important role in the overall quality of candidate drug molecules, because compounds with optimal lipophilicity (logD ≈ 1–3) may have increased chances of success in drug development (Waring, 2010).

The steric, electrostatic, and lipophilic properties of both the ligand and the target-binding site determine the stability of the subsequent ligand-macromolecule complex and the success of the proposed synthetic modifications (Gohlke, 2012). Hydrogen bonds are electrostatic attractions between two dipoles, where the proton covalently bonded to the electronegative donor group carries a positive partial charge and is thus capable of forming a dipole-dipole interaction with the non-bonded pair of electrons of an electronegative atom named acceptor. The hydrogen bonding has been generally described as the largest contributor to the stability of the ligand-macromolecule complex and as being crucial for molecular recognition. These interactions contribute 3–5 kcal/mol/bond in most cases, and the bond strength is dependent on the local medium, particularly the dielectric constant of the surrounding environment, the distance between donor and acceptor, and the direction of the interaction (Warshel et al., 1995). All of these components can be modeled with the aid of a computer for the proposition of novel structures. Another important interaction is the ionic bonds that result from the attraction of two oppositely charged atoms, which are considered long-range interactions (Chang, 2000), which is likely responsible for the initial recognition of the ligand and receptor. The bond strength, which varies between 5 and 10 kcal/mol, is inversely proportional to the distance between the two charged atoms and the dielectric constant of the surrounding medium. These interactions are particularly important due to the ionization of both acids and bases at physiological pH. The optimal van der Waals (VDW) forces (dipole-dipole, dipole induced-dipole, and London dispersion) are dependent on the shape complementarity of the protein active site and the interacting ligand. These interactions result from the attraction between permanent or temporary dipoles produced by the uneven distribution of electron density (Chang, 2000). These short-range interactions are weaker than both ionic and hydrogen bonds with an energy of 0.5–1 kcal/mol. If surface complementarity is high, these interactions can be numerous and thus contribute significantly to the target-ligand complex stability. Likewise, hydrophobic interactions, which occur between two non-polar regions, are also critical for the ligand-macromolecule complexation driven by an increase in entropy (Ladbury et al., 2010). Other non-covalent interactions, such as Π-stacking, halogens bonds, CH-Π, Cation-Π, and complexation with metal ions, can be designed based on experimental X-ray data (Blokzijl and Engberts, 1993).

**Figure 7:**
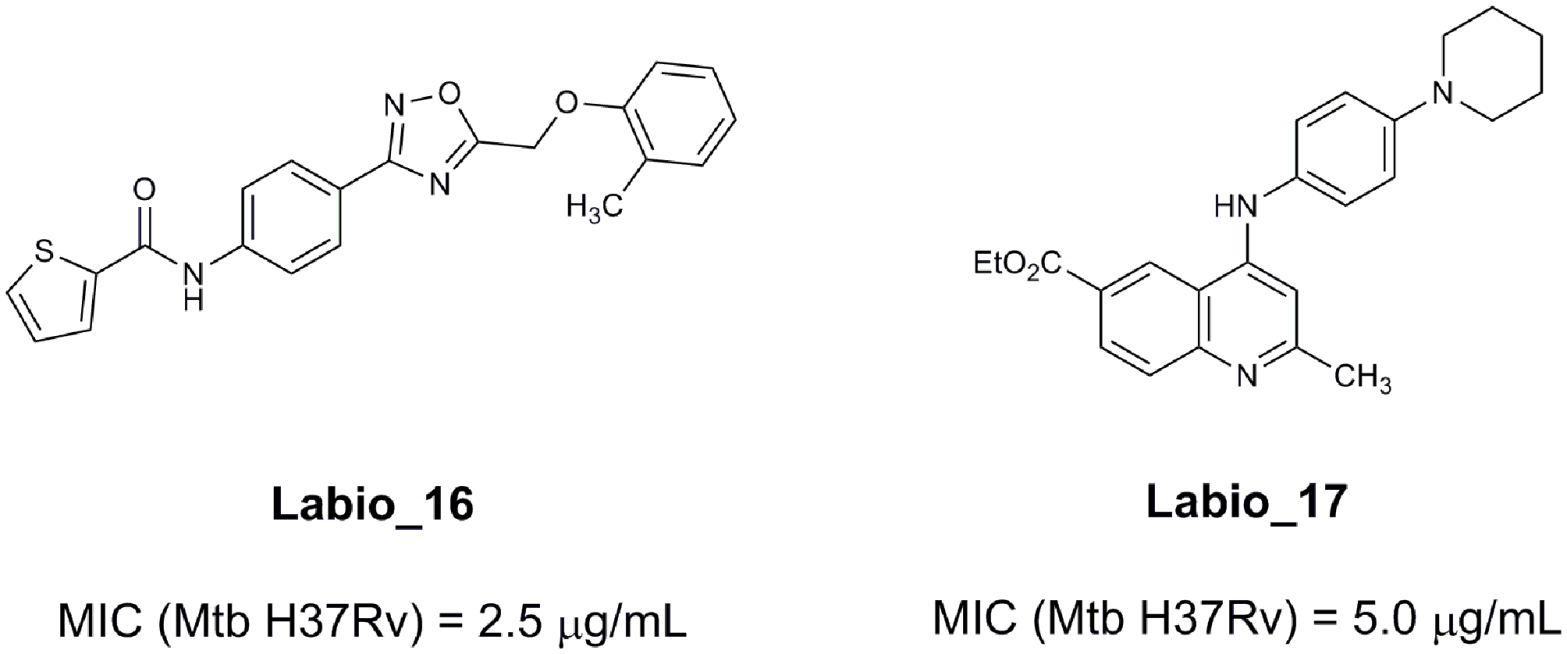
Chemical structure of compounds **Labio_16** and **Labio_17**.

An example of computer-assisted construction of a 3-D pharmacophore model based on available X-ray structural data has been reported for *M. tuberculosis* InhA enzyme (*Mt*InhA), the bona fide target of isoniazid (Pauli et al., 2013). This 3-D pharmacophore served as a basis for virtual screening to identify *Mt*InhA ligands from a library of chemical compounds of the ZINC database. Nineteen molecules from an initial data set of ~1 million that could bind to *Mt*InhA:NADH binary complex were identified (Pauli et al., 2013). The inhibition constants and mode of action were determined by steady-state kinetics for seven of these compounds that inhibited *Mt*InhA activity (Martinelli et al., 2017). The thermodynamic signatures of non-covalent interactions were determined by protein fluorescence spectroscopy and van't Hoff analyses of ligands binding to MtInhA:NADH binary complex. Five new chemotypes inhibited *in vitro M. tuberculosis* H37Rv growth (Martinelli et al., 2017). Two of them, named **Labio_16** and **Labio_17** (Figure 7), also inhibited the *in vitro* growth of PE-003 multidrug-resistant strain. In addition, in Zebrafish model **Labio_16** showed no cardiotoxicity whereas **Labio_17** showed dose-dependent cardiotoxicity. A model was built for the *Mt*InhA:NADH:Labio_16 ternary complex (Martinelli et al., 2017). Crystal structure determination of this ternary complex are currently underway to provide experimental data on which to base medicinal chemistry efforts on this likely hit for the development of chemotherapeutic agents to treat TB.

A computer-aided screening workflow has been described that combines knowledge-based bias with chemical space diversity to guide the design of screening sets that is expanded so that is applicable to any collection of molecules (Jansen et al., 2019). These authors identify “quality” starting points that should increase the chances of success. The “quality” criteria are unique for each project and attributes such as physical chemical property profile (e.g., low lipophilicity, high ligand efficiency), lack of activity in counter-assays (e.g., cytotoxicity assays), confirmation in orthogonal assays, and novelty of chemotype should be taken into account (Jansen et al., 2019). The concept of doing an integrated design in which the diversity set complements the focused set(s) within the same design run prevents sampling the same chemical space twice (Jansen et al., 2019). The workflow put forward by Jansen et al. (2019) has been named Biased Complement Diversity (BCD) and has been made available. Basically, initial screening set selection by employing BCD workflow uses clustering to describe chemical space and then applies the bias by picking the “highest quality” compounds per cluster (Jansen et al., 2019). It may be worthwhile to try the BCD workflow to find initial hits for later assessment of inhibitory activity on *M. tuberculosis* growth as users can decide on the preferred method of clustering and fingerprints. Interestingly, the BCD workflow is applicable to collections of commercially available compounds or virtual compound collections. Quality attributes and exclusions are project-specific annotations. Exclusions may be based on undesirable physical chemical properties, undesirable sub-structures, and evidence from prior screening history that a compound is a frequent hitter (e.g., PAINS). For instance, the BCD workflow may be employed to identify a novel chemical matter using a known and validated mechanism of TB pathogenesis.

Pathogens present a range of challenges for penetration and intracellular accumulation of drug candidates. For intracellular pathogens, an additional barrier is the requirement to enter the eukaryotic host cell to reach the biochemical target of a drug candidate. The characteristics of compounds that can pass through cell wall and cell membranes are hard to predict. Computational models to predict the physicochemical properties that dictate intracellular accumulation of low molecular mass chemical compounds have been proposed to aid in the discovery and development of antibiotics against Gram-negative bacteria (Richter et al., 2017). These properties could be used in SAR studies to develop compound series into drug candidates. The predictive guidelines for accumulation in *E. coli* (eNTRy) state that compounds are most likely to accumulate if they contain a non-sterically encumbered ionizable nitrogen atom (primary amines are the best), have low three-dimensionality (globularity ≤ 0.25), and are relatively rigid (rotatable bonds ≤ 5) (Richter et al., 2017; Richter and Hergenrother, 2019). However, whether this model may yield useful anti-TB drug candidates remains to be demonstrated.

## Recent Alternatives for Tb Therapy

**Figure 8:**
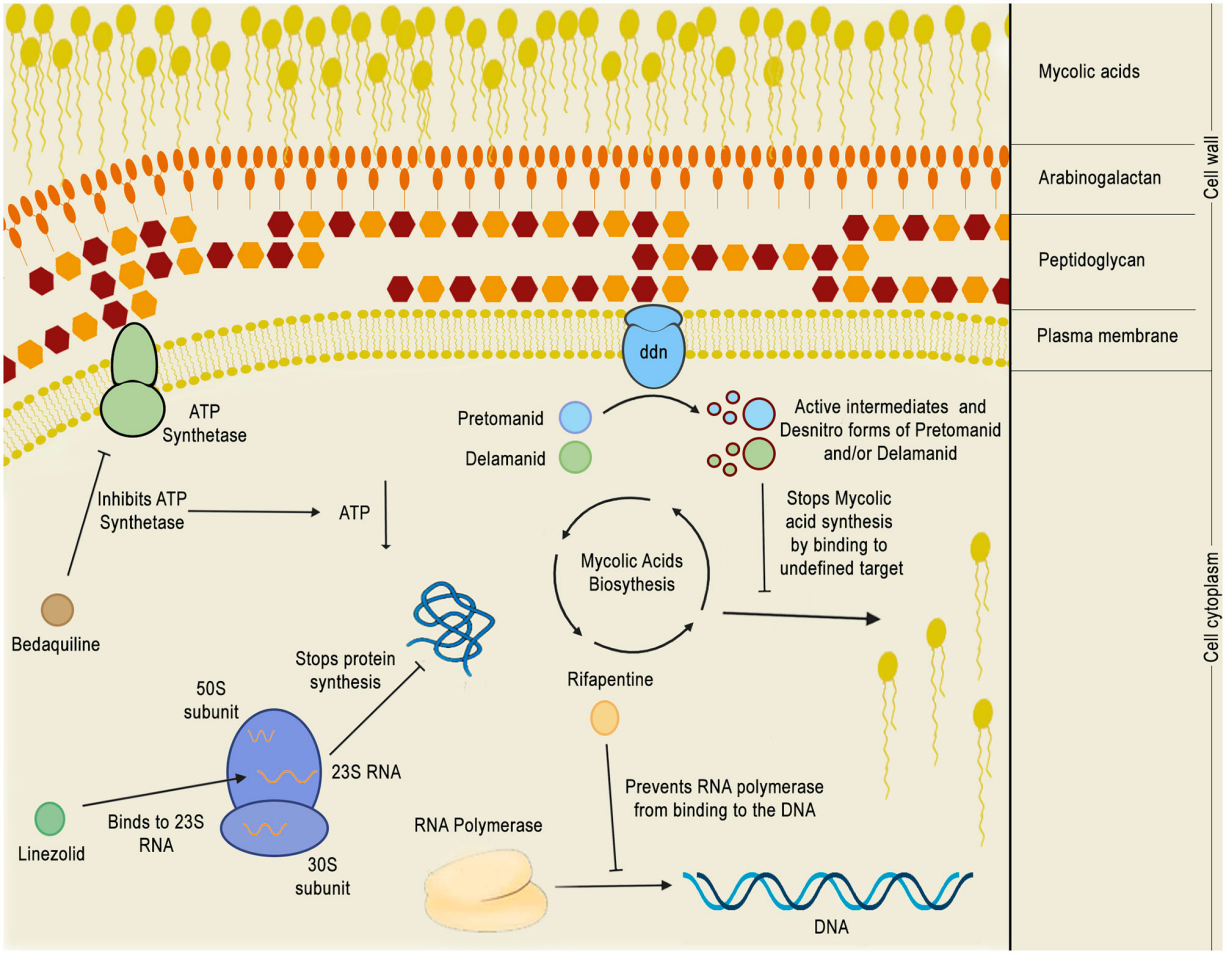
Mechanism of action of phase 3 anti-TB drugs. The figure depicts the mechanism of action of five phase 3 drugs inside the bacillus cytoplasm: bedaquiline, pretomanid, delamanid, rifapentine, and linezolid. Bedaquiline depletes the intracellular energy storage by inhibiting the mycobacterial ATP synthetase which, in turn, results in a reduction of ATP production. Linezolid acts by binding to 23S RNA in the ribosome 50S subunit, inhibiting translation. Rifapentine binds to the RNA polymerase, preventing it from binding to the DNA and shutting down bacterial transcription. Pretomanid and delamanid are pro-drugs that are converted into their bioactive forms by deazaflavin dependent nitroreductase (ddn), which is thought to be a membrane protein. Pretomanid or delamanid active intermediates that are formed during the reaction are able to bind to undefined targets of the mycolic acid biosynthetic pathway, inhibiting the formation of mycolic acids, which are crucial for the mycobacterial cell wall.

Despite the great difficulty in obtaining new compounds with all necessary characteristics for the success of TB treatment, the use and combination of different approaches for the identification of anti-TB compounds have proven to be fruitful in recent years, as many compounds have reached phase III clinical trials and thus are promising alternatives to diminish the burden of TB. In total, 21 potential drugs are at present in clinical trials as an alternative for treating drug-susceptible, drug-resistant, and latent tuberculosis (World Health Organization, 2019). These compounds include both newly-discovered molecules exhibiting anti-tubercular activity and FDA-approved drugs, originally aimed for treating other diseases, whose use has been redirected for TB therapy after a drug repurposing process. All of these compounds are presented in Table 1, along with their chemical structure, chemical class, mechanism of action and the strategies that led to their discovery. The mechanism of action of the phase III drugs bedaquiline, pretomanid, delamanid, rifapentine, e linezolid are illustrated in Figure 8.

**Table 1:** Overview of ongoing anti-TB drugs in clinical trials.

**Drug/inhibitor**	**Drug class**	**Strategy of identification**	**Mechanism of action**	**References**
**Drugs in phase 3 trials**				
Bedaquiline 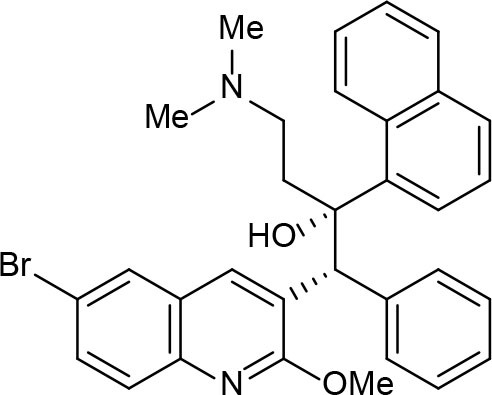	Diarylquinoline	Whole-cell screenings	Inhibits mycobacterial ATP synthase and depletes cellular energy stores	Andries et al., 2005
Delamanid 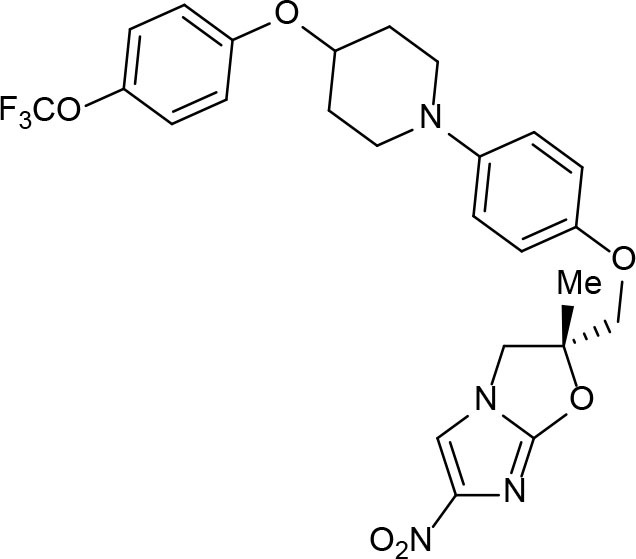	Nitroimidazole	Screening compounds that could inhibit the mycolic acid biosynthesis, followed by assays related to safety and efficacy, and then SAR studies	Inhibits mycolic acids biosynthesis	Fujiwara et al., 2018
Pretomanid 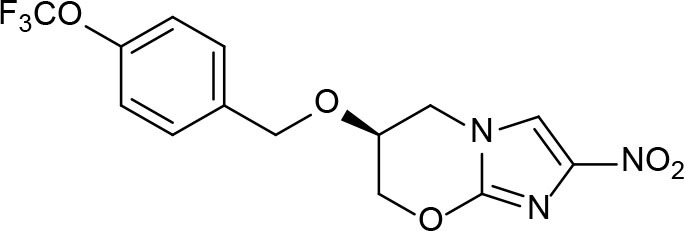	Nitroimidazole	SAR studies of nitroimidazole	Inhibits mycolic acid biosynthesis	Barry et al., 2004; Haver et al., 2015
Clofazimine 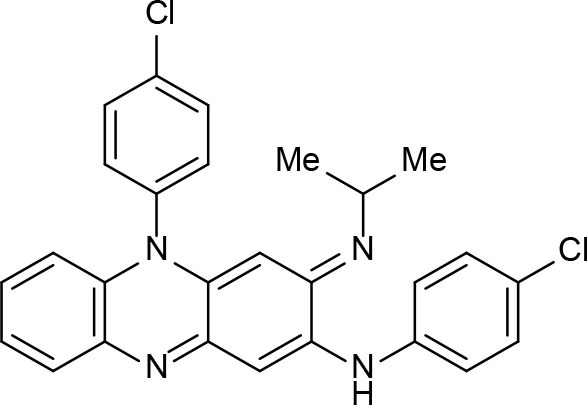	Riminophenazine	Drug repurposing	Presumably competes with menaquinone (MK-4), a key cofactor in the mycobacterial electron transfer chain	Yano et al., 2011
Rifapentine^*^ 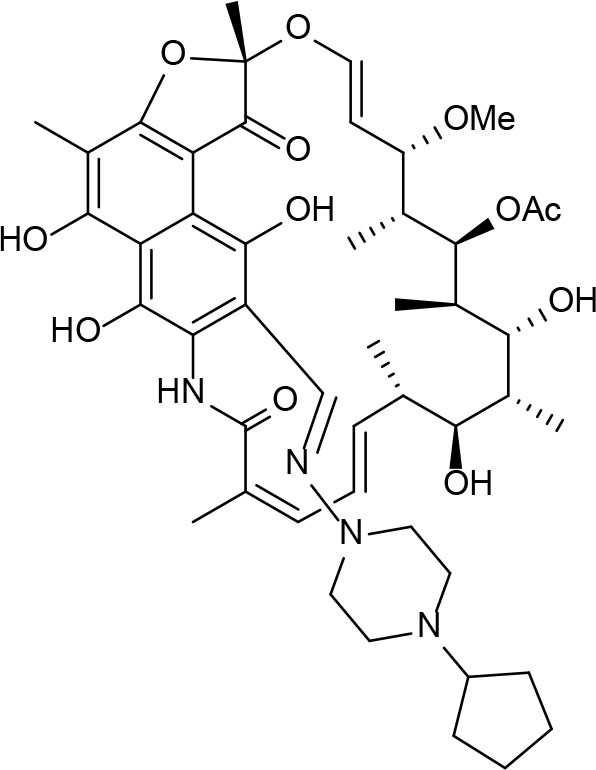	Macrolactam	SAR studies of rifampin	Inhibits bacterial RNA polymerase	Dorman et al., 2020
Moxifloxacin^*^ 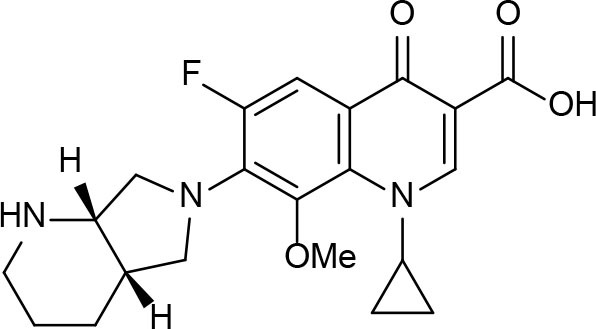	Fluoroquinolone	Drug repurposing	Inhibits DNA synthesis by binding to DNA gyrase	Schedletzky et al., 1999
Linezolid 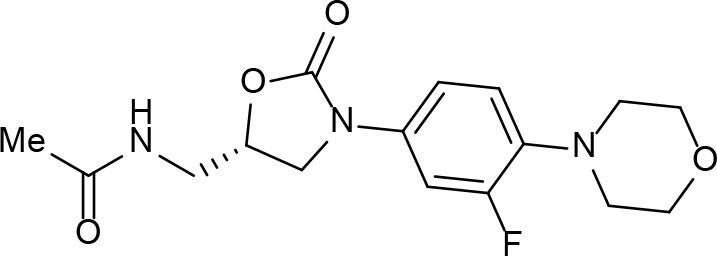	Oxazolidinone	Drug repurposing	Inhibits protein synthesis by binding to 23S RNA in the 50S ribosomal subunit of bacteria	Mukhtar and Wright, 2005
**Drugs in phase 2 trials**				
Telacebec (Q203) 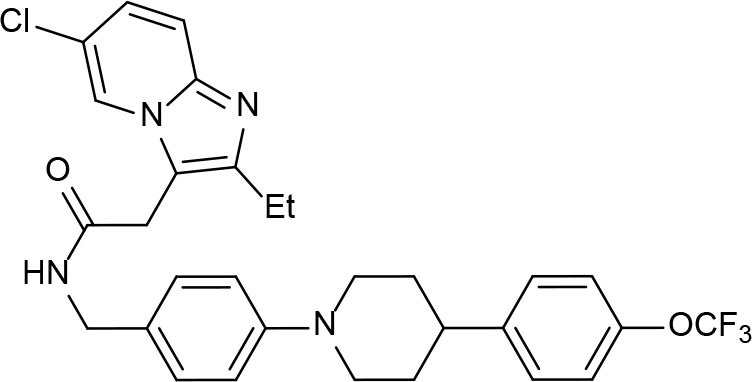	Imidazopyridine	Screenings in infected macrophages	Inhibits the cytochrome *bc1* complex and disrupts the electron transport chain for ATP synthesis	Pethe et al., 2013
Delpazolid 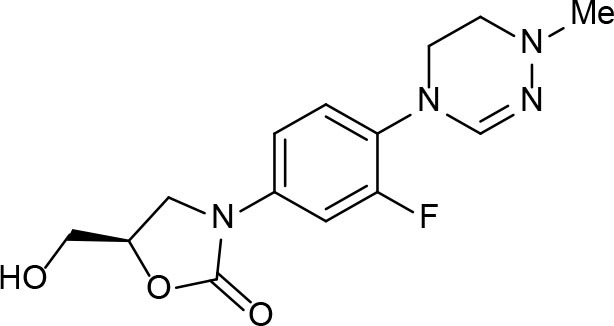	Oxazolidinone	SAR study of oxazolidinone	Inhibits proteins biosynthesis by binding to domain V of 23S rRNA	Jeong et al., 2010
SQ109 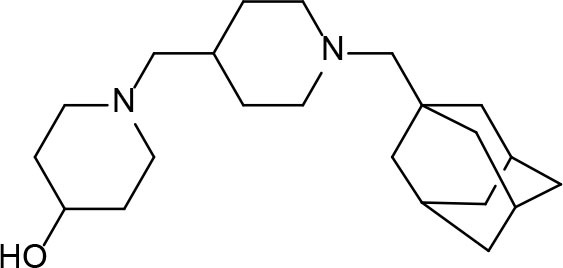	Diethulene dianine	Whole-cell screenings	Acts by targeting MmpL3, a transmembrane protein that transports trehalose monomycolate for cell wall synthesis	Protopopova et al., 2005; Tahlan et al., 2012
Sutezolid 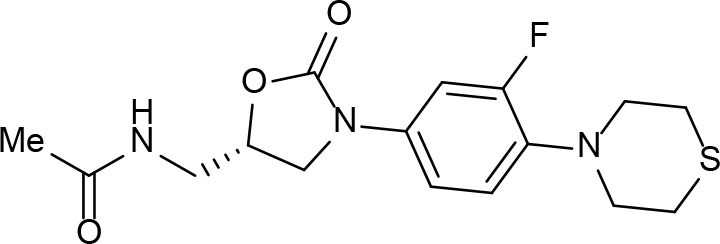	Oxazolidinone	SAR studies of oxazolidinone	Inhibits protein synthesis by binding to 23S RNA in the 50S ribosomal subunit of bacteria	Wallis et al., 2014
Nitazoxanide 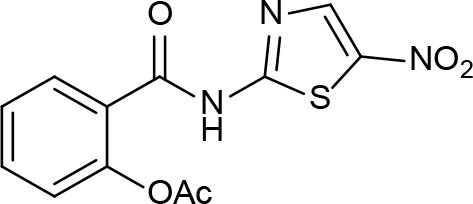	Thiazolides	Drug repurposing	Inhibits pyruvate-ferredoxin oxidoreductase, nitroreductases and peptide disulfide isomerases	de Carvalho et al., 2009
**Drugs in phase 1 trials**				
BTZ-043 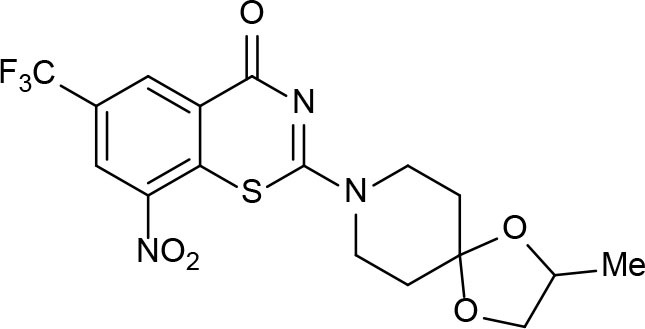	Benzothiazinone	Whole-cell screenings	Inhibits decaprenyl-phosphoribose epimerase (DprE1)—cell wall biosynthesis	Makarov et al., 2009; Lechartier et al., 2012
GSK-3036656 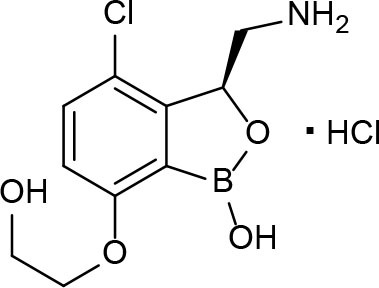	Oxaborole	SAR studies of 3-aminomethyl 4-halogen benzoxaborole	Inhibits protein synthesis by binding to leucyl-tRNA synthetase	Li et al., 2017
Macozinone (PBTZ169) 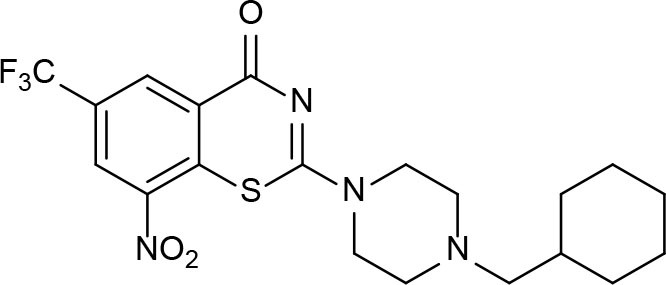	Benzothiazinone	Whole-cell screenings	Inhibits decaprenyl-phosphoribose epimerase (DprE1)—cell wall biosynthesis	Makarov et al., 2009, 2014
OPC-167832 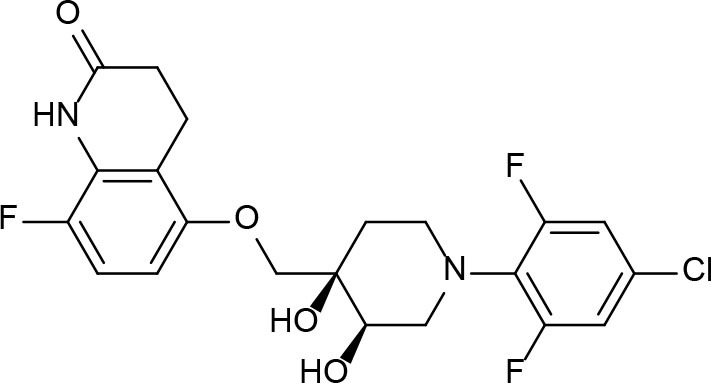	Carbostyril	SAR studies of carbostyril	Inhibits decaprenyl-phosphoribose epimerase (DprE1)—cell wall biosynthesis	Hariguchi et al., 2020
SRP720 (VXc-486) 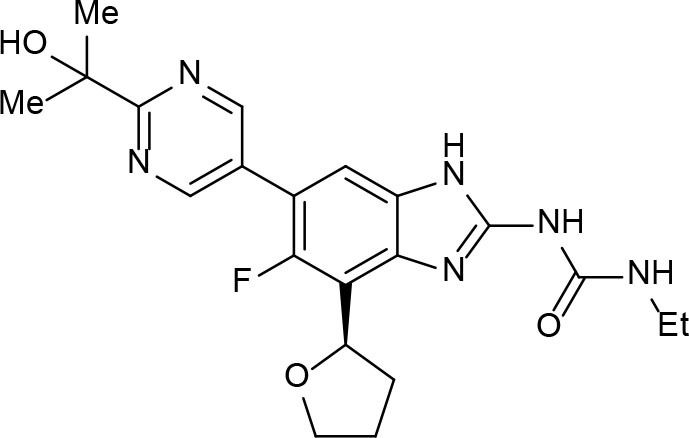	Aminobenzimidazole	SAR studies of aminobenzimidazole	Inhibits DNA synthesis by binding to DNA gyrase	Locher et al., 2015
TBA-7371 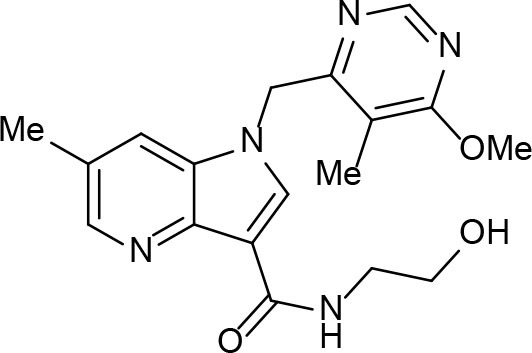	1,4-Azaindoles	Scaffold morphing strategy followed by lead optimization	Inhibits decaprenyl-phosphoribose epimerase (DprE1)—cell wall biosynthesis	Shirude et al., 2013, 2014
Contezolid (MRX-1) 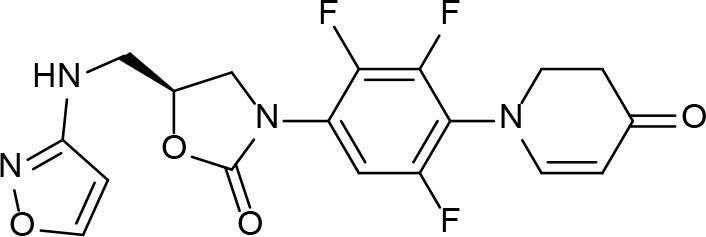	Oxazolidinone	SAR studies of oxazolidinone	Inhibits protein synthesis by binding to 23S RNA in the 50S ribosomal subunit of bacteria	Gordeev and Yuan, 2014
TBI-166 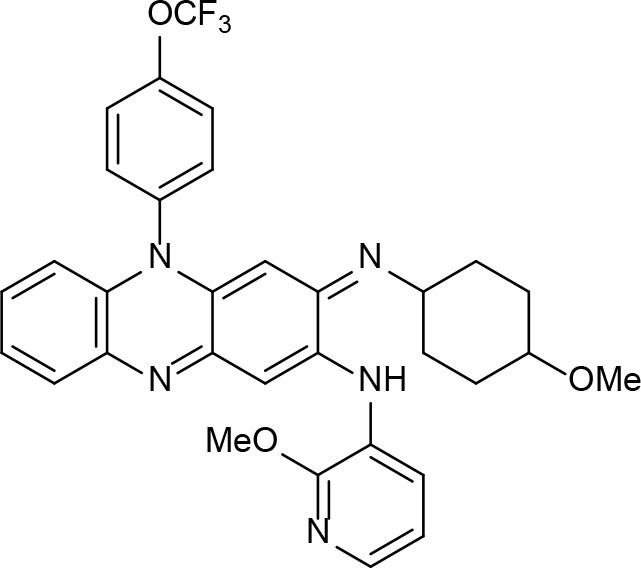	Riminophenazine	SAR studies of rimonophenazine	Probably the same mechanism as clofazimine	Lu et al., 2011; Xu et al., 2019
TBI-223 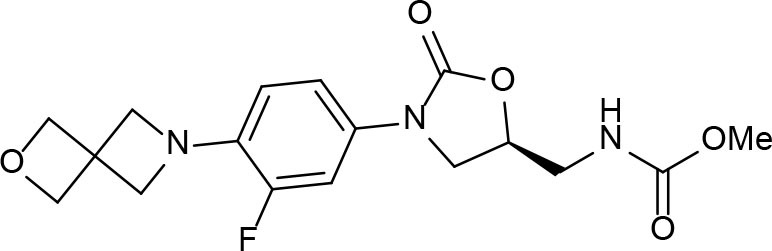	Oxazolidinone	SAR studies of oxazolidinone	Inhibits protein synthesis by binding to 23S RNA in the 50S ribosomal subunit of bacteria	https://www.tballiance.org/portfolio/trial/12012

* *Drugs already used against TB, but that are presented in phase 2 or 3 studies, which may be due to their combination with different drugs or in different doses*.

Bedaquiline (Table 1), a diarylquinoline compound, was approved, in 2012, by the Food and Drug Administration (FDA) through an accelerated process for use in MDR-TB (Cox and Laessig, 2014). It was in 2005 that a classic phenotypic screening of different chemical series was tested against *M. smegmatis* and a chemical optimization of the lead compound led to this potent inhibitor of *M. tuberculosis* (Andries et al., 2005). After 15 years, it is still undergoing clinical trials, although it is being used in the treatment of drug-resistant TB. Bedaquiline has a mechanism of action that differs from those currently used drugs: it inhibits mycobacterial ATP synthase and depletes cellular energy stores (Andries et al., 2005). In phase-2 clinical trials, it was shown that patients treated with bedaquiline-combined therapy achieved a faster sputum culture conversion (SCC) (sputum must be free of *M. tuberculosis*) than a placebo combination therapy (Diacon et al., 2009). Unfortunately, bedaquiline treatment group suffered an increased overall mortality. A study to evaluate the safety and tolerability, as well as efficacy of bedaquiline, was performed in 2014 (Diacon et al., 2014). The overall mortality of the drug was lower when compared with the previous study (Diacon et al., 2009) and the most common adverse effects were similar to those frequently reported in other MDR-TB therapies, although an effect on cardiac electrophysiology (QTcF prolongation) was identified in some patients (Diacon et al., 2014; Pontali et al., 2017). For now, bedaquiline should only be prescribed when there is no other alternative for treating MDR-TB (Tiberi et al., 2017).

Delamanid (Table 1) is another compound from a novel antibiotic class to be conditionally approved. It was approved in 2014 by the European Medicines Agency and should only be used when an effective treatment regimen cannot be prescribed due to resistance or tolerability issues. The first step for the identification of delamanid as an anti-TB was to seek for new agents in literature, compounds library and commercially available compounds (Liu et al., 2018). Compounds from various drug classes that inhibit mycolic acid synthesis were synthesized and evaluated for their safety and efficacy, which led to identification of the nitroimidazole class compounds (Liu et al., 2018). As previous studies on bicyclic nitroimidazooxazole compound with favorable *in vitro* activity and *in vivo* efficacy were discontinued due its mutagenic potential, chemical derivatization and SAR studies aimed at reducing mutagenic liabilities and improved antimycobacterial activity of nitroimidazol compounds were carried out, which finally led to the selection of delamanid (Liu et al., 2018). Delamanid is a nitroimidazooxazine that inhibits mycolic acid biosynthesis, preventing the synthesis of ketomycolic and methoxymycolic acids (Blair and Scott, 2015). It is a prodrug that requires intracellular bioreduction of its nitro group generating reactive species that are toxic to the cells. Spontaneous strains resistant to delamanid presented mutations in at least one of five genes (*dnn, fgd1, fbiA, fbiB*, and *fbiC*), whose products participate in an F420-dependent nitroreductase pathway and likely play a role in the metabolism of delamanid to its active form. At the present time, it can be only speculated that some unidentified active intermediates generated during the bioactivation can exert antimycobacterial activity (Fujiwara et al., 2018). In 2019, it was published the randomized, double-blind, placebo-controlled, phase 3 trial study, whose aim was to evaluate the safety and efficacy of delamanid in the first 6 months of treatment in patients with MDR-TB. Unfortunately, the efficacy was not as expected: no difference in the median time to sputum culture conversion was identified between placebo or delamanid group and the results were discrepant in comparison to the phase 2 scheme (von Groote-Bidlingmaier et al., 2019). The authors suggested that over-performance of the placebo group may have underpowered the phase 3 trial, which, however, added certainty in the safety but discrepancy in terms of efficacy of delamanid. All patients in this trial received a pre-treatment for 90 days prior to enrolment. However, since delamanid has an early and immediate effect, as noted in 2 months treatment in the randomized phase 2 trial, this pre-treatment could result in a delamanid underperformance (von Groote-Bidlingmaier et al., 2019). However, even in the context of those external factors, a consistent and more favorable outcome was seen in the primary, sensitivity and subgroup analyses. In addition, patients treated with delamanid had lower proportions of acquired resistance (von Groote-Bidlingmaier et al., 2019). The safety results of this trial showed that delamanid is well-tolerated and is consistent with the phase 2 scheme. Like bedaquiline, the main concern is the QTcF prolongation effect that was lower in this study than previously observed in phase 2 clinical trials (von Groote-Bidlingmaier et al., 2019).

Pretomanid (Table 1) is a nitroimidazooxazine antimycobacterial agent as delamanid, and share the same mechanisms of activation and action (Haver et al., 2015). Interestingly, an untargeted metabolomics study of pretomanid treatment in *M. smegmatis* may have revealed a new mode of action of this compound: an accumulation of phosphate-sugars was linked to a toxic metabolite methylglyoxal that showed antimicrobial activity (Baptista et al., 2018). Drug regimens with bedaquiline, pretomanid and linezolid (BPaL), and bedaquiline, pretomanid, moxifloxacin, and pyrazinamide (BPaMZ), have been developed to treat drug-susceptible and drug-resistant TB (Keam, 2019). The former achieved a higher cure rate in 6 months of treatment in patients with MDR-TB or XDR-TB (Keam, 2019). The latter demonstrated superior antimycobacterial activity when compared to a group treated with isoniazid, rifampicin, pyrazinamide, and ethambutol against drug-resistant TB (Tweed et al., 2019). FDA has approved the combination of BPaL for the treatment XDR-TB.

Reporpusing or repositioning of FDA-approved drugs, as interactions with unexpected targets are possible, should shorten the length of time invested in developing novel antibiotics due to the already-proven drugs' safety (Maitra et al., 2015). Some drugs that have been through this process have already been proved to be effective for treating MDR- and XDR-TB (Wallis et al., 2014). The edaravone drug, which is FDA-approved for treating amyotrophic lateral sclerosis and stroke, was shown to inhibit the production of reactive oxygen species. It slowed down the development of mutations in bacterial cultures without losing its ability to kill bacteria, preventing the rapidly evolving “gambler” cell subpopulation from raising (Pribis et al., 2019). However, whether edaravone can slow the evolution of drug resistant strains, with or without antibiotic treatment, in models of TB infections, remains to be shown. Levofloxacin, gatifloxacin and moxifloxacin are all fluoroquinolones whose use has been redirected for treating isoniazid-resistant TB and MDR-TB (Guglielmetti et al., 2016). Despite their potential for reducing the overall duration of TB therapy, results of some clinical trials have not been consistent with that, suggesting that a combination of these drugs might be inferior to the standard regimen (Jawahar et al., 2013; Gillespie et al., 2014). Moreover, resistance to fluoroquinolones may also arise as an obstacle for prolonged therapy. As suggested by Horsburgh et al. (2015), bedaquiline could be used as a substitute for fluoroquinolones after resistance has been set. Clofazimine, which is effective for treating leprosy—caused by *Mycobacterium leprae*—has been suggested to be useful for TB therapy as well, and has undergone many clinical trials, with an overall success rate of 61% (Dey et al., 2013).

Many other compounds are currently on their way for repurposing, ranging from anthelminthic (Lim et al., 2013) to alcohol withdrawal drugs (Horita et al., 2012). The usage of chemical systems biology approach allowed Kinnings et al. (2009) to explore further the function of entacapone and tolcapone, formerly used for treating Parkinson's disease mainly. After an *in silico* proteome-wide analysis, they predicted the enzyme InhA to be an off-target for these drugs, which was confirmed *in vitro* by kinetic assays. Nitazoxanide, used in the treatment of infections caused by protozoans like *Giardia* and *Cryptosporidium*, is a prodrug whose mechanism of action is thought to consist in entering cells and inhibiting pyruvate-ferredoxin oxidoreductase, nitroreductases, and peptide disulfide isomerases (de Carvalho et al., 2009). It is now a promising alternative for treating TB, as its antimycobacterial activity has recently been unveiled. What increases the appeal of this compound is also the lack of reports of resistant bacterial strains during clinical trials after exposure to the drug (de Carvalho et al., 2009). Owing to the clear potential of drug repurposing for finding alternatives for TB treatment in a short period of time, approaches such as the one developed by Battah et al. (2019), which combines *in silico* high-throughput docking and *in vitro* phenotypic screenings altogether, and the one proposed by Kinnings et al. (2009), have been developed to provide researchers an alternative for the most common drug discovery pipelines (Farha and Brown, 2019).

## Parting Thoughts

The withdrawal of large pharma companies from the development of new antimicrobial field represents a major challenge to combat infectious diseases (Parish, 2019). The lack of return on investment for antibiotics and the difficulties in obtaining regulatory approval are among the reasons for pharma withdrawal from the field. Another consequence of pharma withdrawal is the loss of a wealth of industry expertise in antibacterial drug development, which cannot be easily replaced (Parish, 2019). Hence, ideally, pharmaceutical companies should be persuaded to contribute to the development of chemotherapeutic agents to treat infectious diseases, including TB. New incentives and initiatives have been launched to drive up interest, including GARDP (Global Antibiotic Research & Development Partnership) and CARB-X (Combating Antibiotic Resistant Bacteria), which fund research and development, as well as incentives such as the Tropical Disease Priority Review Voucher Program, which can be applied to TB (Parish, 2019). The establishment of the Global Antimicrobial Resistance Research and Development Hub is also a valuable initiative of the German government by providing 500 million euros of investment (Parish, 2019). However, whether these types of initiatives will prove to be sufficient to provide economic and scientific stimuli to generate a robust pipeline of antimicrobials remain to be seen.

The Lancet Commission on Tuberculosis reported a critical reflection on progress to-date and a roadmap for countries and their development partners to achieve global commitments toward ending the TB epidemic (Reid et al., 2019). This report stresses that declines in TB mortality are not keeping pace with reductions in deaths from other infectious diseases of global importance such as HIV and malaria, and the world is not on track to meet targets set out in the Sustainable Development Goals and the WHO End TB Strategy (Reid et al., 2019). The Commission report also notes that the WHO's End TB Strategy target of reducing TB mortality by 90% by 2030 will not be reachable without a substantial increase in global TB R&D investment, from $772 million per year in 2017 to at least $2 billion per year during the next 4 years (Reid et al., 2019). The return on investment of each dollar spent could range from $16–82 as new tools are produced (Reid et al., 2019). The recommendations of the Lancet Commission on Tuberculosis include investment in TB research and development of therapeutics to overcome the challenge of tuberculosis and HIV co-infection (Reid et al., 2019). Finding novel targets or targets that do not have cross-resistance with current therapeutic drugs should be pursued to achieve better outcomes (Reid et al., 2019). As pointed out by Reid et al. (2019), “two major challenges in developing novel, safer, shorter, and simpler regimens are the research costs of preclinical development and phase 1 and 2 clinical trials, and the lack of reliable, validated biomarkers that can be used to predict the duration of therapy necessary to cure virtually all patients treated with a given therapy.” A diversified portfolio of therapeutic products offers the best hope for long-term success in developing a 2-month universal regimen against all forms of TB; however, substantial investment in the short-to-medium term is needed to guarantee those products reach the market (Reid et al., 2019).

We, the authors, have striven to give the readership of Frontiers in Chemistry journal a broad overview of the many hurdles that ought to be overcome to translate a hit for anti-TB candidate (bench top) to a useful chemotherapeutic agent to treat TB (bed side). The various approaches, including, but limited to, medicinal chemistry, screening strategies, experimental tools for assessing desirable features of targets and chemical compounds, new and promising opportunities to TB therapy, and recent findings that may have bearings on TB research have been described. Last, it should be pointed out that host-directed therapy has not been discussed here to try to provide a focused contribution to the readers. Those interested in host-directed therapy, valuable contributions have been given by, for instance, Tiberi et al. (2018), Baindara (2019), and Shim et al. (2020).

## Author Contributions

PM, CB, and LB conceived and wrote the first draft of the manuscript. PD and ES made the table and added information to the manuscript. RR, AR, CN, and FM made figures and added information to the manuscript. BA added information to the text, formatted, and revised the final version for submission. All authors contributed to the article and approved the submitted version.

## Conflict of Interest

The authors declare that the research was conducted in the absence of any commercial or financial relationships that could be construed as a potential conflict of interest.
